# The size distribution of nanoparticles emitted from advanced manufacturing devices impacts predicted carcinogenic potential

**DOI:** 10.3389/fpubh.2025.1582690

**Published:** 2025-04-09

**Authors:** W. Cary Hill, Andrey Korchevskiy

**Affiliations:** ^1^ITA International, LLC, Blacksburg, VA, United States; ^2^Chemistry and Industrial Hygiene, Inc., Arvada, CO, United States

**Keywords:** 3D printing, particulates, carcinogenic potential, excess lung cancer risk, PM2.5, PM10, advanced manufacturing

## Abstract

Advanced manufacturing devices such as 3D printers bring users into closer contact with processes that generate ultrafine particles or release engineered nanomaterials. While approaches to assessing the risk of lung carcinogenesis and related health effects are developing, serious questions exist regarding the impact such devices may have on human health and safety if proper actions (i.e., engineering controls including ventilation or filtration) are not taken to mitigate exposures. The size distribution of particulates emitted during fused deposition modeling (FDM) 3D printing was measured following the ANSI/CAN/UL 2904 method and associated lung cancer risk was estimated through a developing model. Particulate morphologies were assessed, identifying agglomerative and morphological characteristics which may further impact health effects. The estimation of excess lung cancer risk for 3D printer emissions based upon particle size was found to vary according to aerodynamic diameter distribution and emitted concentration, with values projected as high as 468 cases per 10,000 workers in the measured exposure scenario (1 m^3^ enclosure with air fully exchanged once per hour); predicted excess lung cancer risk was found to drop significantly as print extrusion temperature decreased. Actual health impacts will depend highly upon the exposure scenario, as room air volume, ventilation, and number of printers in operation will impact the concentration of particulates present. This model provides a means for assessing excess lung cancer risk across a broad aerodynamic diameter distribution, improving resolution over methods that use a single particle size bin such as PM2.5 or PM10. The effects of particle composition are only anecdotally considered in this model, however; this limitation should be accommodated as the model is implemented in practical settings.

## 1 Introduction

Fused deposition modeling (FDM) fabrication, or 3D printing, provides innovators and hobbyists with a means for rapid prototyping and physical production of creative designs using inexpensive polymeric feedstocks. The relative low cost of these 3D printers and related polymeric materials represents a low barrier to entry, promoting rapid growth in sectors which traditionally exhibit limited environmental health and safety oversight such as startups and in personal spaces (1). Accordingly, risks associated with use may not be appropriately monitored nor mitigated by engineering controls, elevating opportunities for user exposure to particulates (2–11) and volatile organic compounds (VOCs) (12–18) released during 3D printing. While a growing body of literature continues to quantify exposures measured during 3D printing in various controlled and deployed scenarios (2–20), assessment of the toxicological context is limited. Studies have reported limited or no acute effects on inflammatory markers after very short exposure durations (i.e., 1 h) (21), but other studies modeling lung deposition of 3D printer emissions have found significant deposition potential in the lower respiratory tract which may lead to chronic health effects (22, 23).

Various toxic effects have been correlated to fine and ultrafine particle exposures (24–30), with toxic effects of nanoparticles (ultrafines) noted to include respiratory effects and systemic reactions including immunosuppression and immunomodulation (31, 32). Particulate exposures are often classified in accordance with standardized size nomenclature, with fine particulates measuring less than 2.5 μm in diameter (PM2.5) receiving significant attention. Prolonged exposure to fine particulates is associated with increased risk of elevated fasting blood glucose and low-density lipoprotein cholesterol levels (33) and lipid changes associated with hypertension (34). Respirable dust (PM10) and fine particulate (PM2.5) exposures are associated with increased mortality and negative effects including lung cancer incidence, cardiovascular disease, and respiratory disease, with effects increasing for smokers and vulnerable groups (35–37).

We previously developed, described, and reported a new methodology for modeling lung cancer risk related to exposure to particulate emissions created during printing of acrylonitrile butadiene styrene (ABS), polylactic acid (PLA) and glycol-modified polyethylene terephthalate (PETG) (38). The proposed methodology was based on several approaches, including a development of a specific “carcinogenic potential” value for fine and ultrafine particles that was demonstrated to be a power-spline function of aerodynamic diameter. In this paper we will use the proposed model for a wider class of filaments and extrusion temperatures while increasing the resolution of size-distributed data to discern diameter-specific contributions of emitted particles to lung cancer risk estimations.

## 2 Materials and methods

### 2.1 Measurement of 3D printer emissions

Particulate emission data was collected during printing of common polymeric FDM filaments on the same 3D printer, a Lulzbot Taz 6 (Fargo Additive Manufacturing Equipment, Fargo, ND, USA), using either a M175 print head (for 1.75 mm filaments) or a SE 0.5 print head (for 2.85 mm filaments). Prints were conducted within an electropolished stainless steel enclosure with internal dimensions of 0.9 m × 0.9 m × 1.2 m per the specifications defined by ANSI/CAN/UL 2904 (39).

The enclosure was positioned in a Class 1000 cleanroom, where all measurements were conducted to ensure that any significant particulate releases were attributable to the printers and filaments being studied rather than ambient airborne dust. Particles measuring 0.01–0.3 μm in diameter were measured using a TSI NanoScan 3910 scanning mobility particle sizer (SMPS), while particles measuring 0.3–25 μm in diameter were quantified using a TSI Aerotrak 9306 optical particle counter (OPC) (TSI, Shoreview, MN, USA). Only particulate emissions were assessed in this study; VOC emissions are also prominent during printing of many filaments and exhibit separate, additional contributions to lung cancer risk and other negative health outcomes (18, 40–42) which are not considered in the model presented in this study.

Particulates were sampled onto track-etched polycarbonate (PCTE) membranes (Sterlitech, Auburn, WA, USA) and mounted in conductive cassettes (Zefon International, Ocala, FL, USA) suspended 0.6 m above the print head using a sampling pump operating at a rate of 30 liters per minute (LPM). PCTE membranes were prepared for analysis by scanning electron microscopy (SEM, FEI Quanta 600) by mounting onto aluminum SEM stubs using carbon tape (Ted Pella, Redding, CA, USA) followed by sputter coating with a platinum-palladium coating (8 nm thick, Leica ACE600). Membrane sampling was conducted during an additional, separate print from those conducted during quantitative measurements to ensure that the additional intake of the sampling pump would not disrupt the 1/h exchange rate prescribed by ANSI/CAN/UL 2904.

Measurements were made in accordance with the procedural guidance of ANSI/CAN/UL 2904. Summarily, a standard cube measuring 40 mm on each side was printed using each tested filament within the stainless steel enclosure with an air exchange rate of one exchange per hour. Printing of the cube required approximately 4 h to complete, with data collection continuing for 2 h after the conclusion of the print to assess particulate concentration decay. Prints for each filament were conducted in triplicate with standard error reported. Printing parameters are generally dependent upon the filament type, but parameters were standardized where possible across filaments to reduce variables that might be associated with outcomes.

For the purposes of converting measured particle concentrations to mass concentrations, particulates emitted during printing are assumed to be comprised of material with the same density as the originating filament (i.e., 1.05 g/cm^3^ when printing with ABS), though it is important to note that previous analyses of captured particulates emitted during 3D printing suggest that compositional alterations may occur during the printing process (2).

### 2.2 Modeling of lung cancer risk

The developed methodology for modeling lung cancer risk presented by exposures to 3D printer emissions is detailed in our previous work (38). Summarily, the scenario ascribes excess lung cancer risk associated with exposure per 1 μg/m^3^ to a worker or hobbyist for 30 years starting at the age of 30 for 3 h per day and 5 days per week, as might be considered plausible for a worker who uses or is co-located with 3D printers in operation for a substantial part of a shift.

The lung cancer risk is estimated as an attributable risk fraction in Equation 1.


(1) $$ARF=\frac{RR-1}{RR}*100\%$$

Where ARF is the attributable risk fraction (%) and RR is the relative risk of lung cancer.

The average risk of dying from lung cancer among the United States population is used as a baseline, reported to be 450 per 10,000 per lifetime (43). Excess risk of lung cancer was determined based on Equation 2:


(2) $$\mathrm{Risk}=\left[\mathrm{Particulate}\;\mathrm{Concentration}\right]*\mathrm{Potency}$$

Where the Particulate Concentration is reported as μg/m^3^ and is segregated by size; Potency corresponds to the excess lung cancer cases per 1 person associated with exposure to 1 μg/m^3^ of particles of this size range.

It should be noted that in this study we conditionally assumed that potency factor for each size group of the particulates can be characterized by a single value. In practice, the nature of particles, including chemical composition, shape, surface charge and other relevant parameters, should be also taken into account. However, as we demonstrated previously, size distribution of particles can be efficiently used to predict lung cancer potency factors for various agents (38). From here we were able to hypothesize that size-specific potency factor in our study could play a role of “central tendency” estimation of potency across various types of mineral or engineered particulates.

Pulmonary deposition fraction was determined using the Multiple-Path Particle Dosimetry (MPPD) model version 3.04 from the US EPA. The human MPPD template was used with the Yeh/Schumm Symmetric model, with default values of 3,300 mL for the functional residual capacity (FRC) and upper respiratory tract (URT) volume of 50 mL. Exposure conditions were calculated for an upright body orientation, assuming 1 mg/m^3^ aerosol concentration, 12 breaths per minute, and a tidal volume of 625 mL. The inspiratory fraction was 0.5, pause fraction was 0, and the breathing scenario was nasal.

Lung cancer potency associated with size-fractionated deposition was tuned using a bimodal pulmonary deposition curve, as has been demonstrated previously to exist as a function of aerodynamic diameter with modes predicted at 0.05 μm and 3 μm (44, 45) or, similarly, at 0.03 μm and 3 or 4 μm (46). It should be noted that clearance of particles is not considered, which may alter the relationship of toxicity between the modes since the dependence of clearance rate on particle diameter is complex (47).

The carcinogenic potential (CP) metric is modeled by the following power-spline equation in Equation 3.


(3) $$CP=A_{i}+B_{i}*AD^{s_{i}},\mathrm{if}\;\mathrm{AD}\min_{i}<\mathrm{AD}<\mathrm{AD}\max_{i}$$

where CP is a unitless value of relative lung cancer potency of particles (assumed to be less than 1); AD is aerodynamic diameter (μm); s_i_ is the polynomial exponent for the interval *i*; *i* is the number of intervals for the spline function; ADmin_i_ and ADmax_i_ are the lower and upper ends of the *ith* interval; A_i_ and B_i_ are coefficients determined by a fitting procedure for each interval for the spline function to reach the bimodal CP peaks and three minima potency values.

Lung cancer risk per 1 μg/m^3^ is assumed to be proportional to CP as follows in Equation 4:


(4) Risk=C∗CP+D

for coefficients C and D.

The mass median aerodynamic diameter (MMAD) and geometric standard deviation (SD) was derived from literature for each type of particle to develop the spline function through Monte Carlo simulation (2,000 sample values). Excel Solver was used for parameter fitting. Statistica 14.0 was used for statistical calculation. Crystal Ball software was used for the Monte Carlo simulation.

## 3 Results

To efficiently illustrate the particulate emission profiles for various filaments, the measured number concentration and converted mass concentration are reported in Table 1 for the subject filaments. Assessments were conducted in triplicate for each filament, and standard error is visualized as the thickness of the plotted lines. Tested filaments were of the “natural” color without particle additives (such as metal particles or glass fiber).

**Table 1:** Particulate concentrations emitted during 3D printing varied widely depending upon filament composition and extrusion temperature.

Filament and extrusion temperature	Number concentration emission profile	Mass concentration emission profile
ABS (220°C)	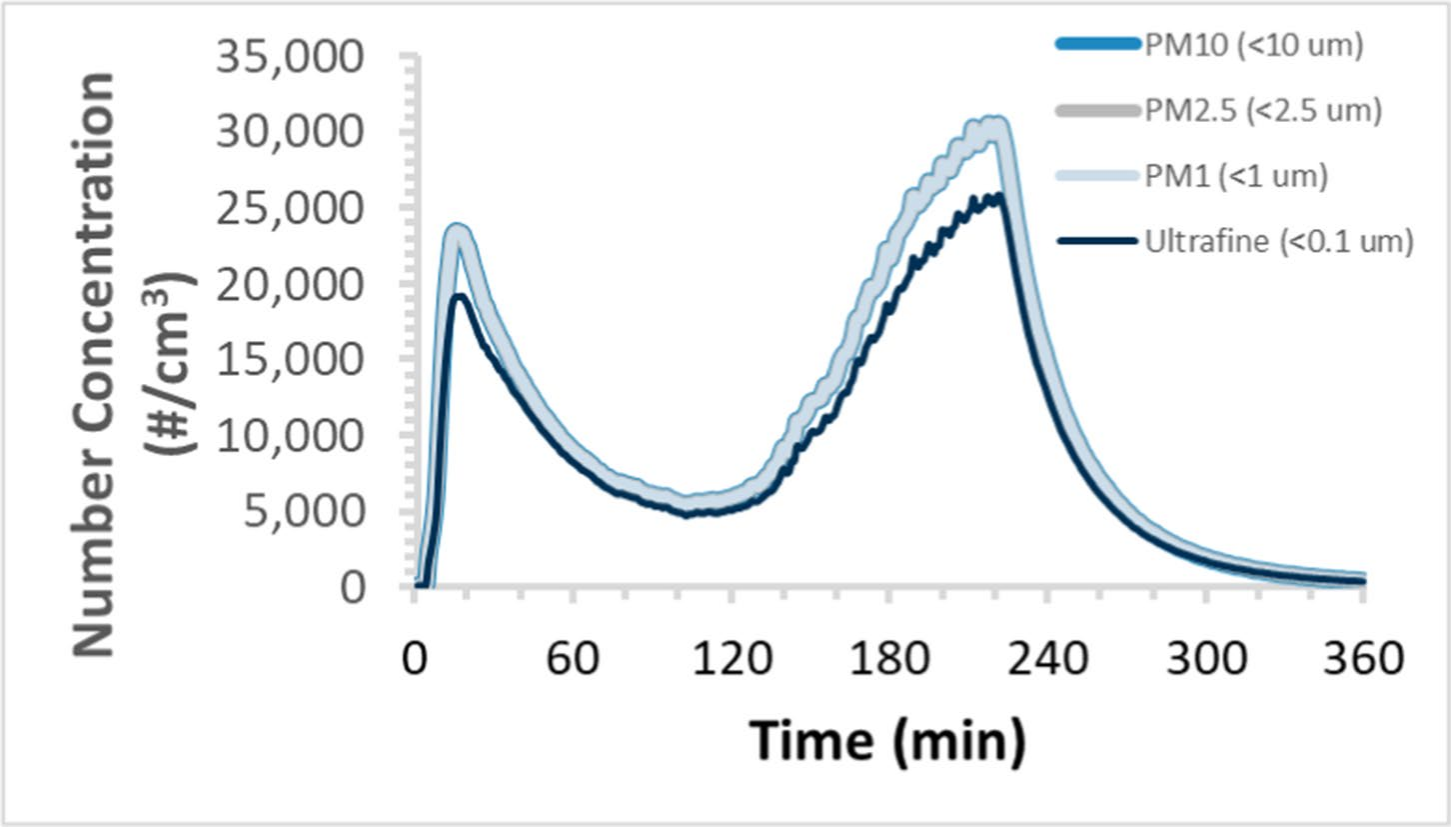	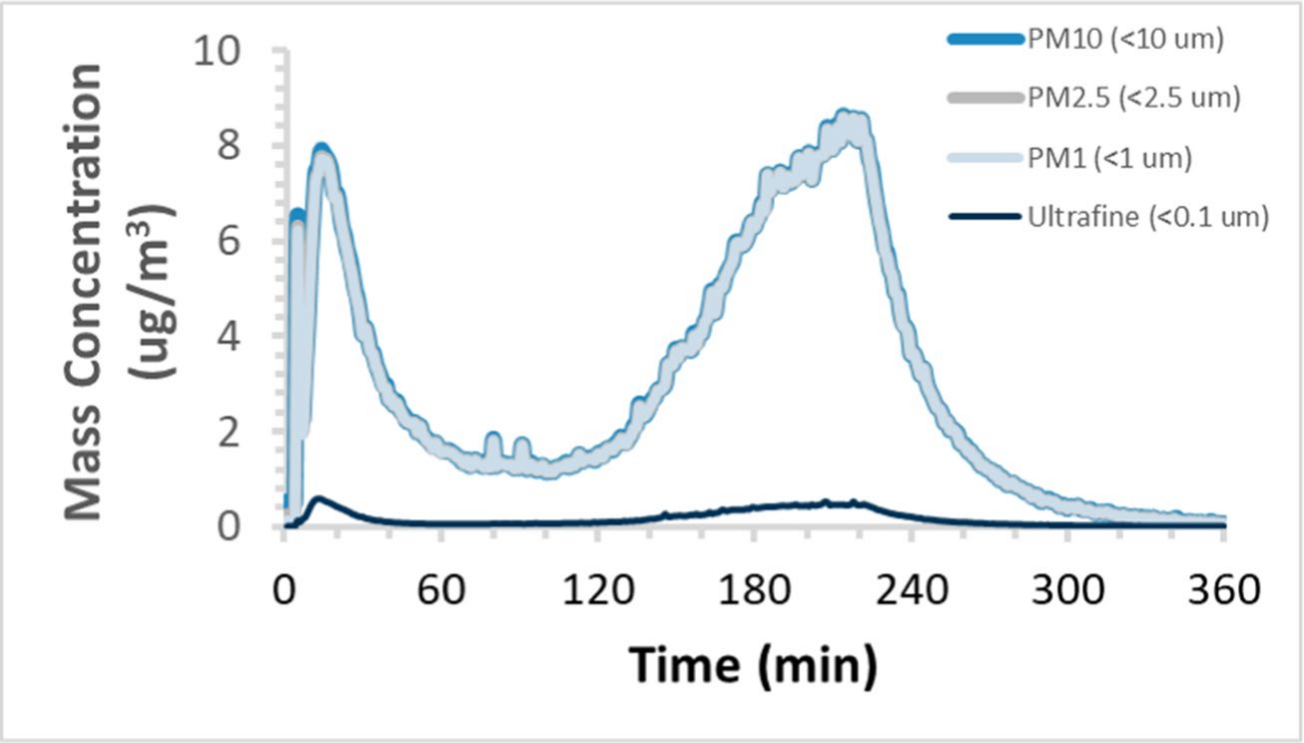
ABS (230°C)	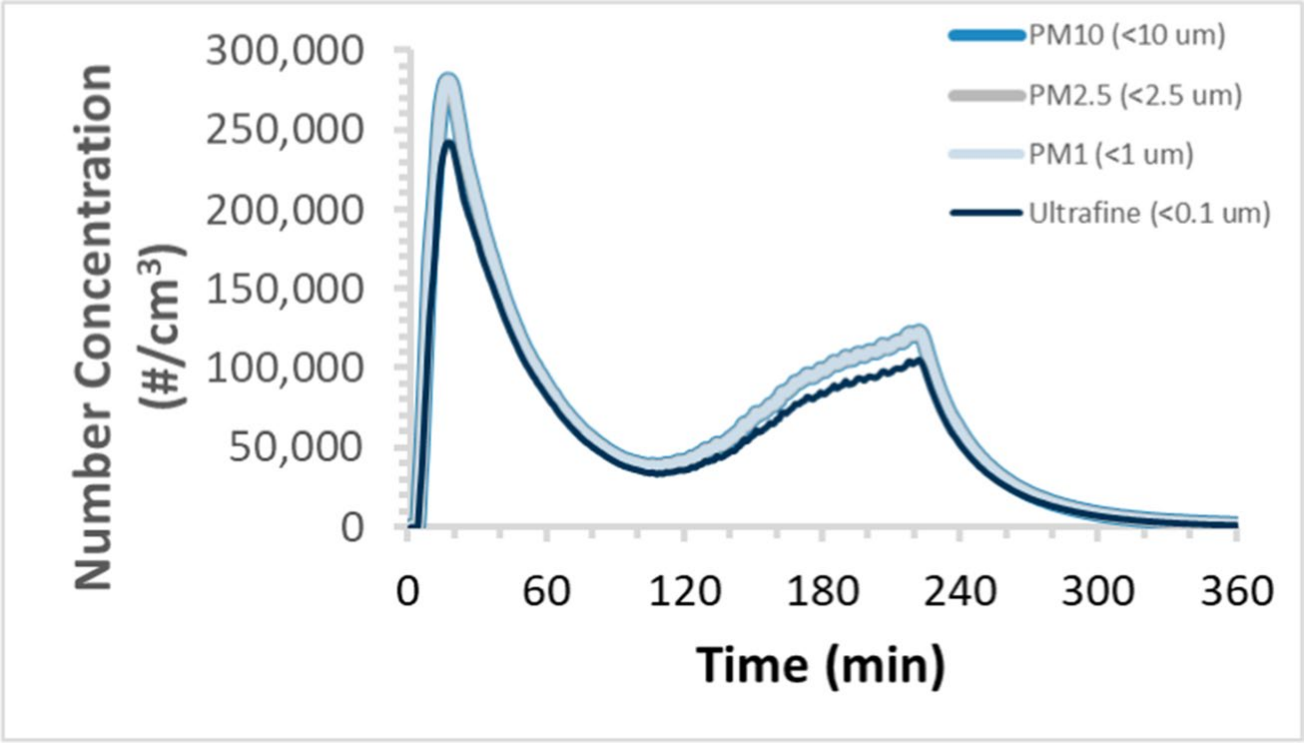	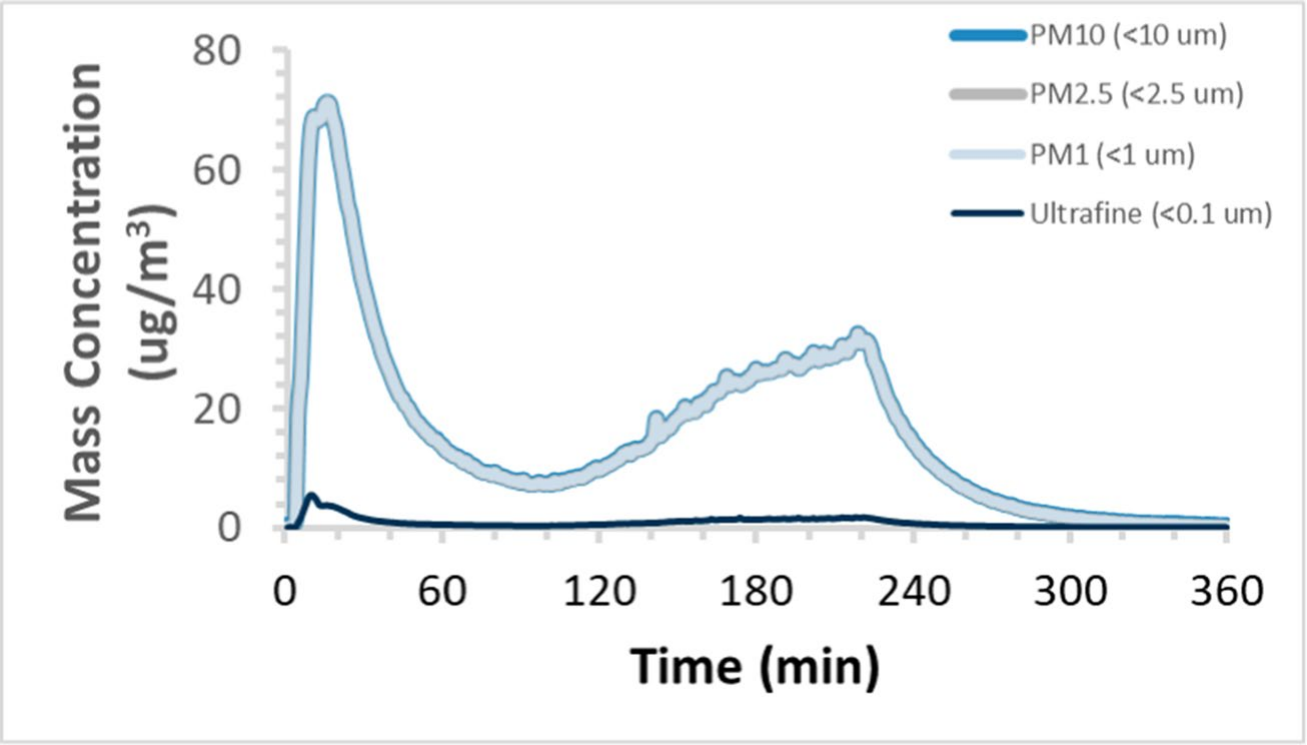
ABS (240°C)	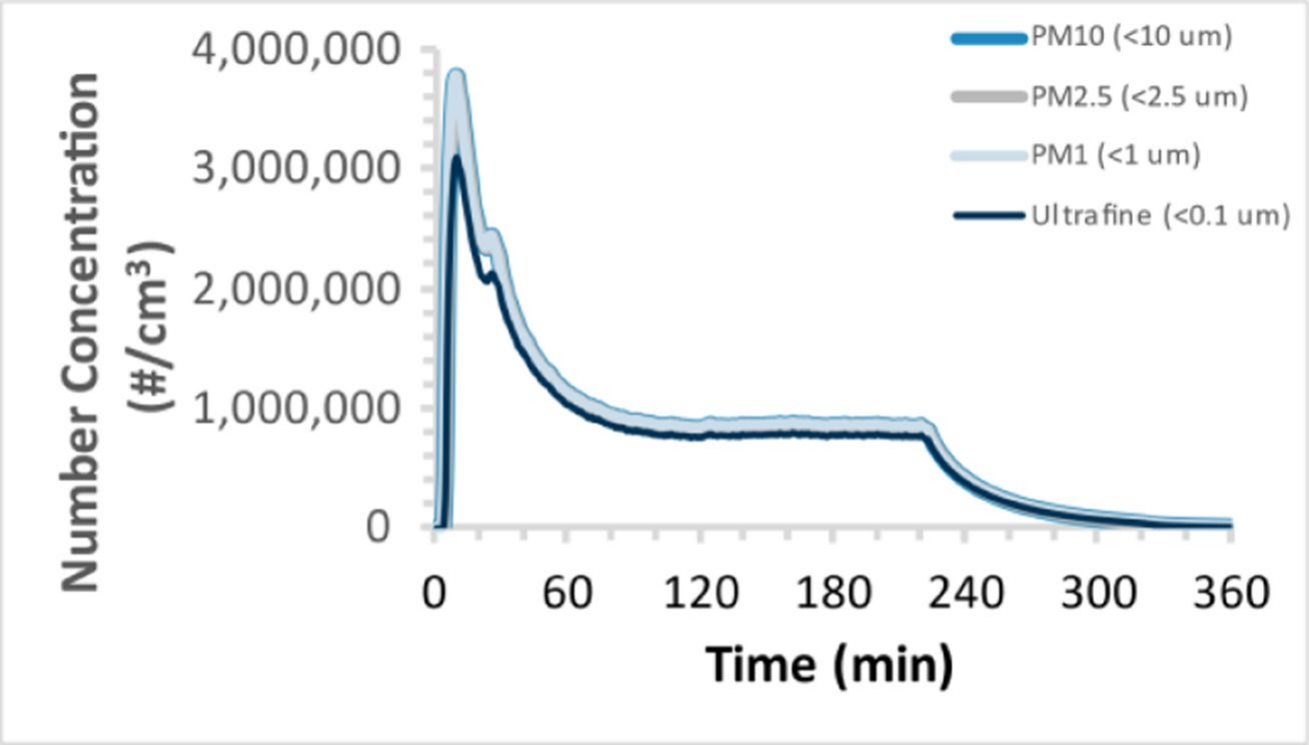	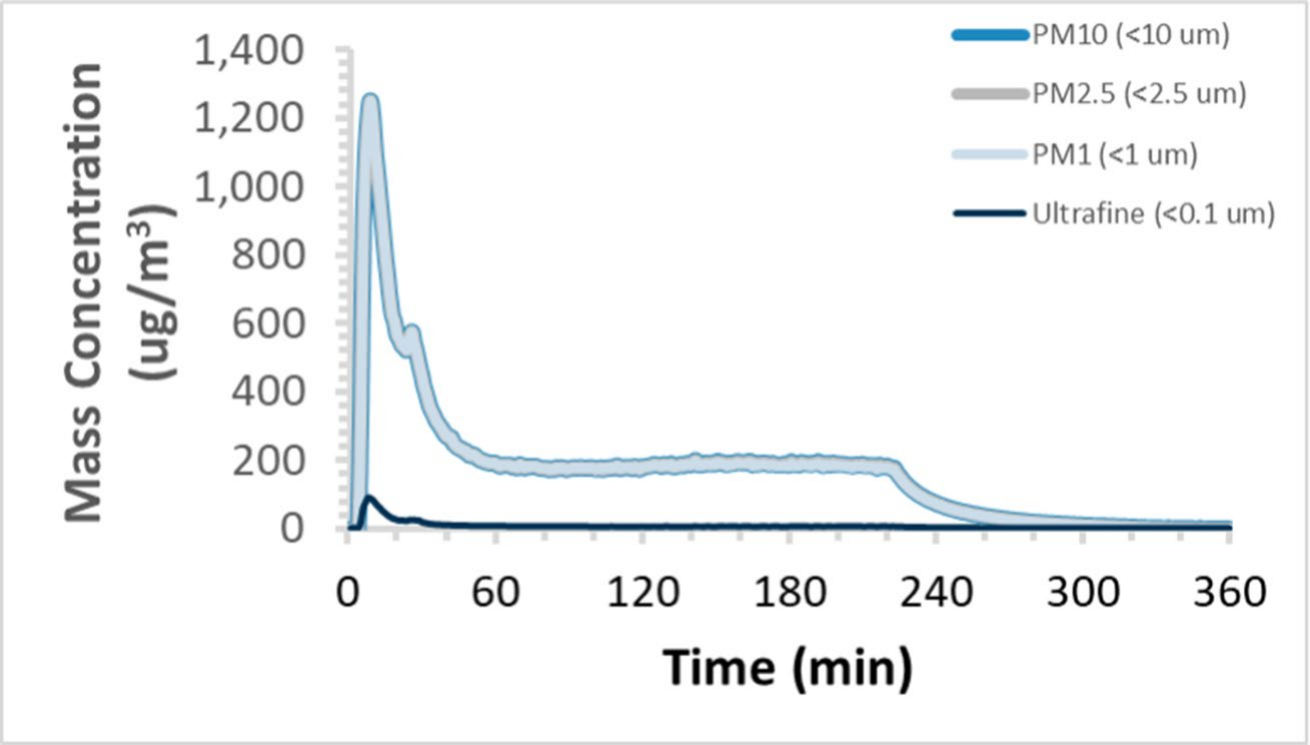
PLA (200°C)	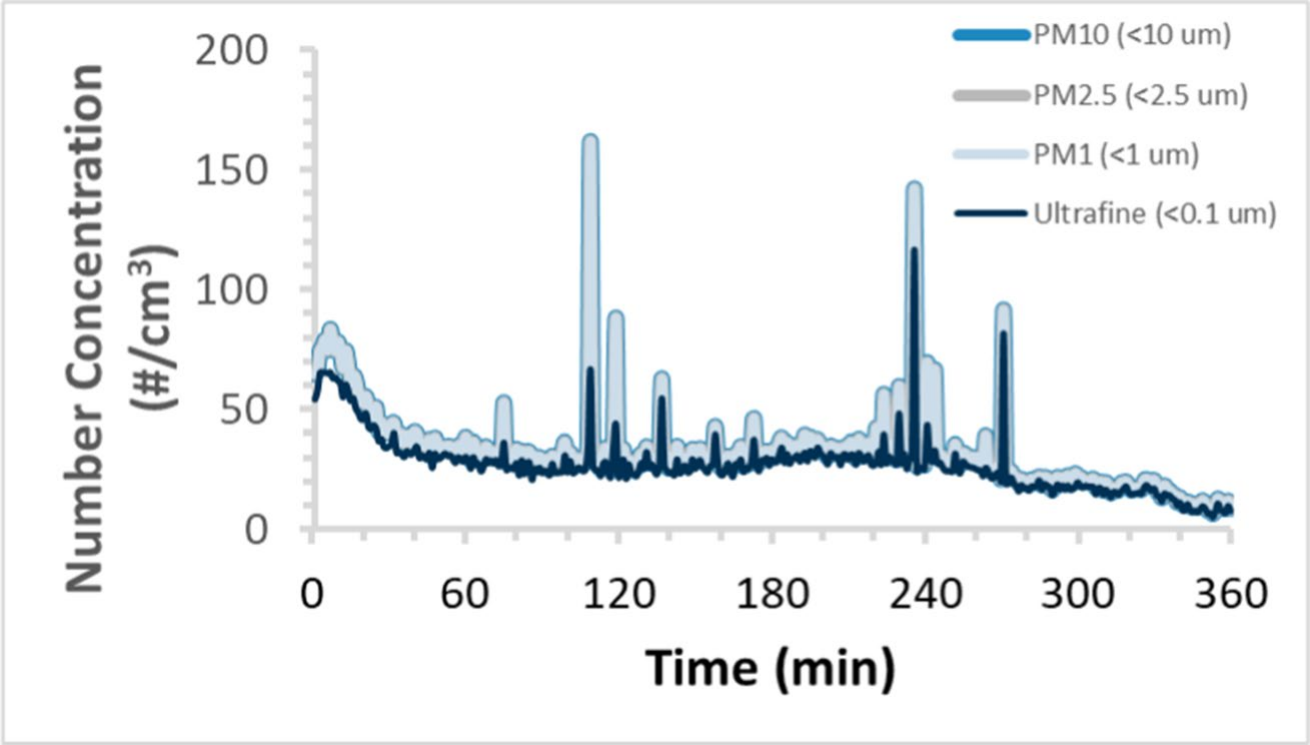	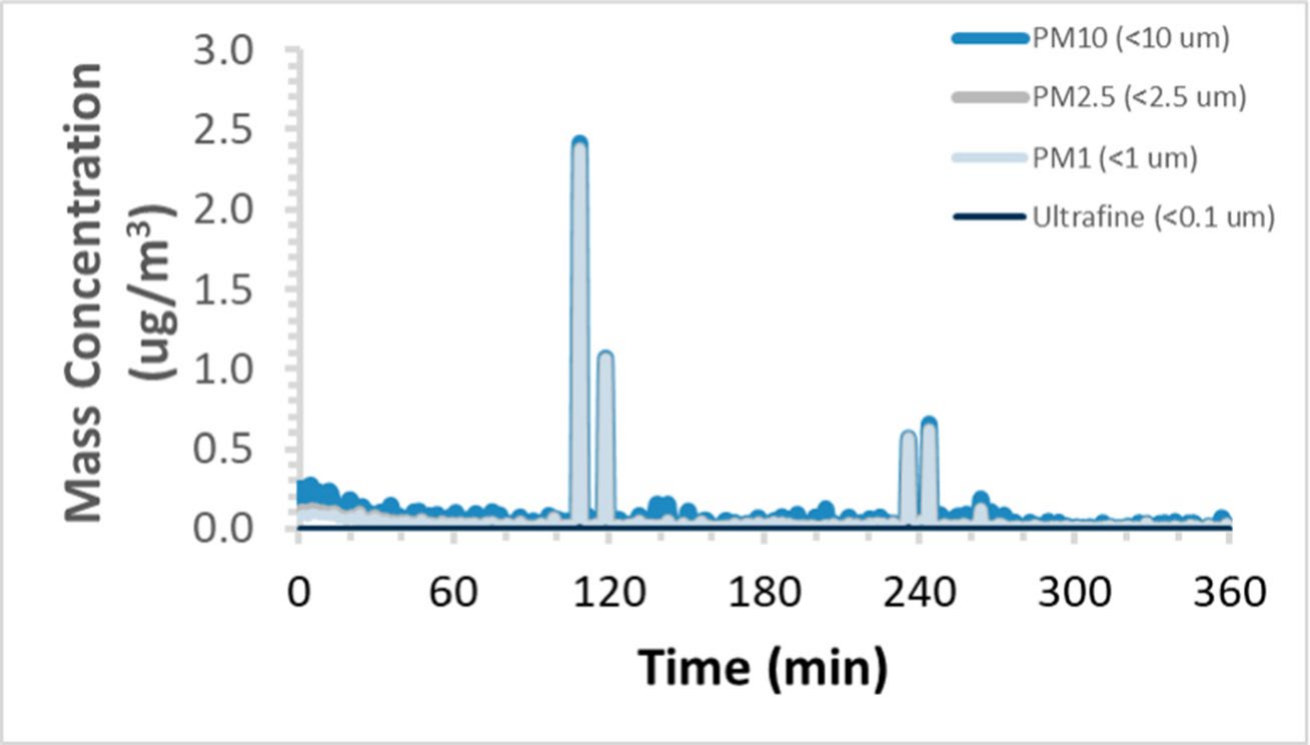
PLA (220°C)	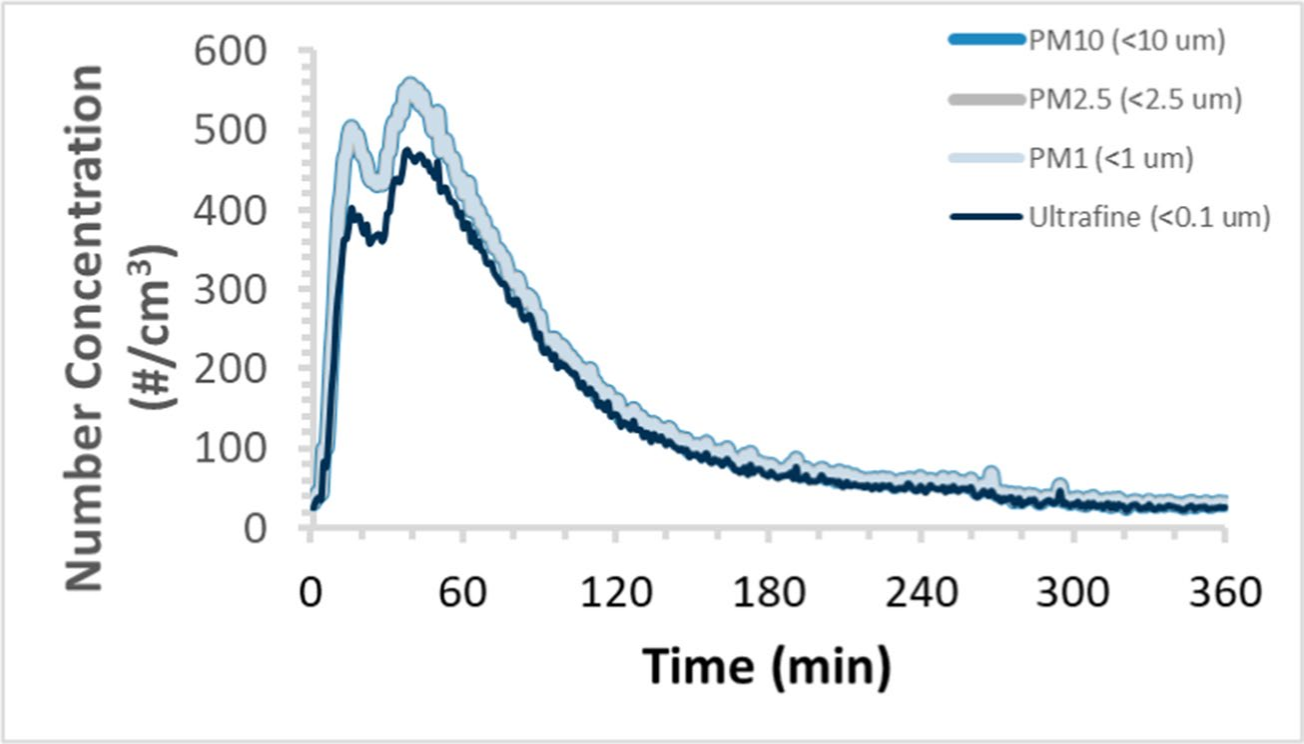	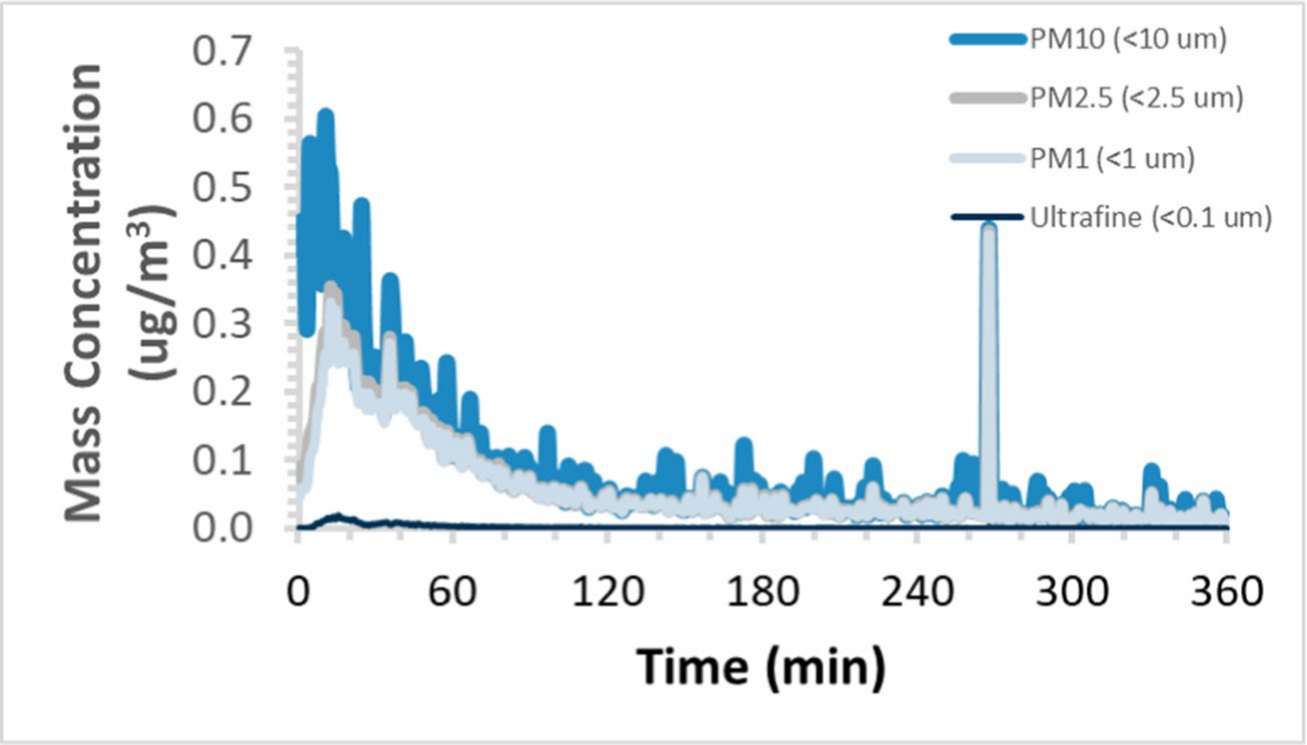
PLA (230°C)	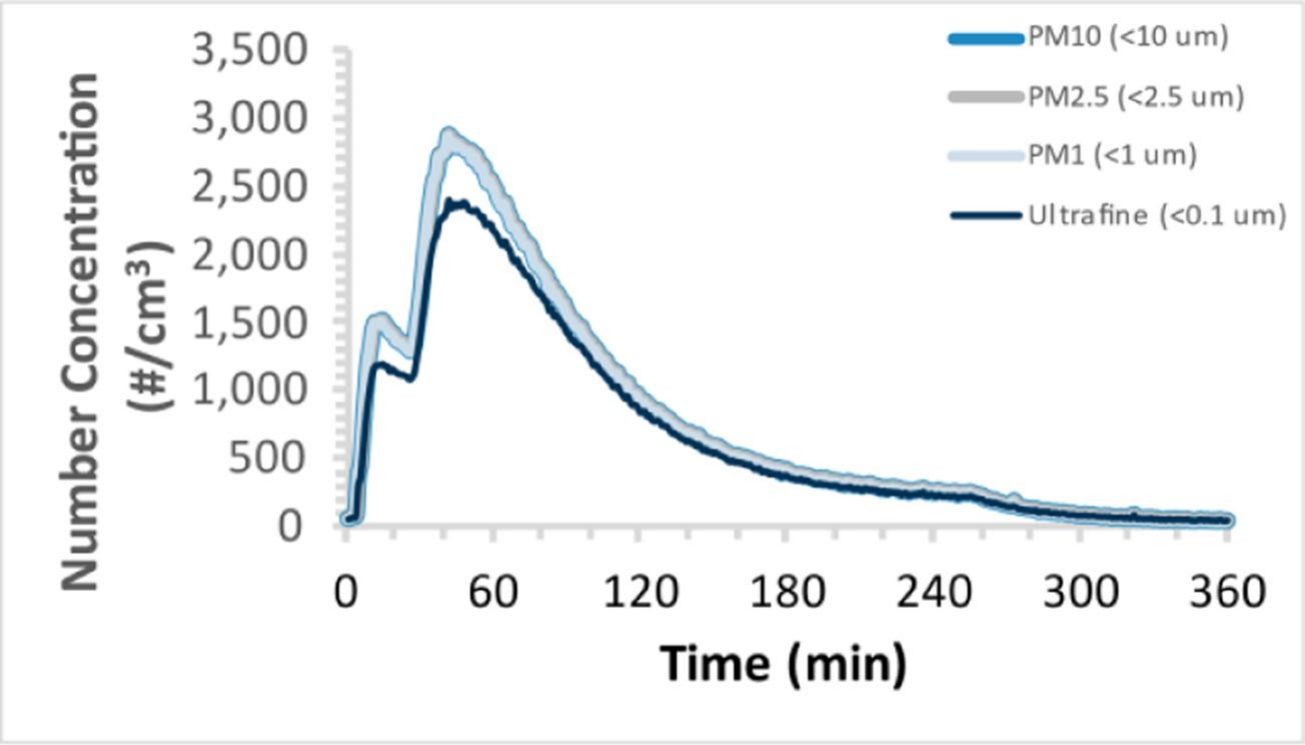	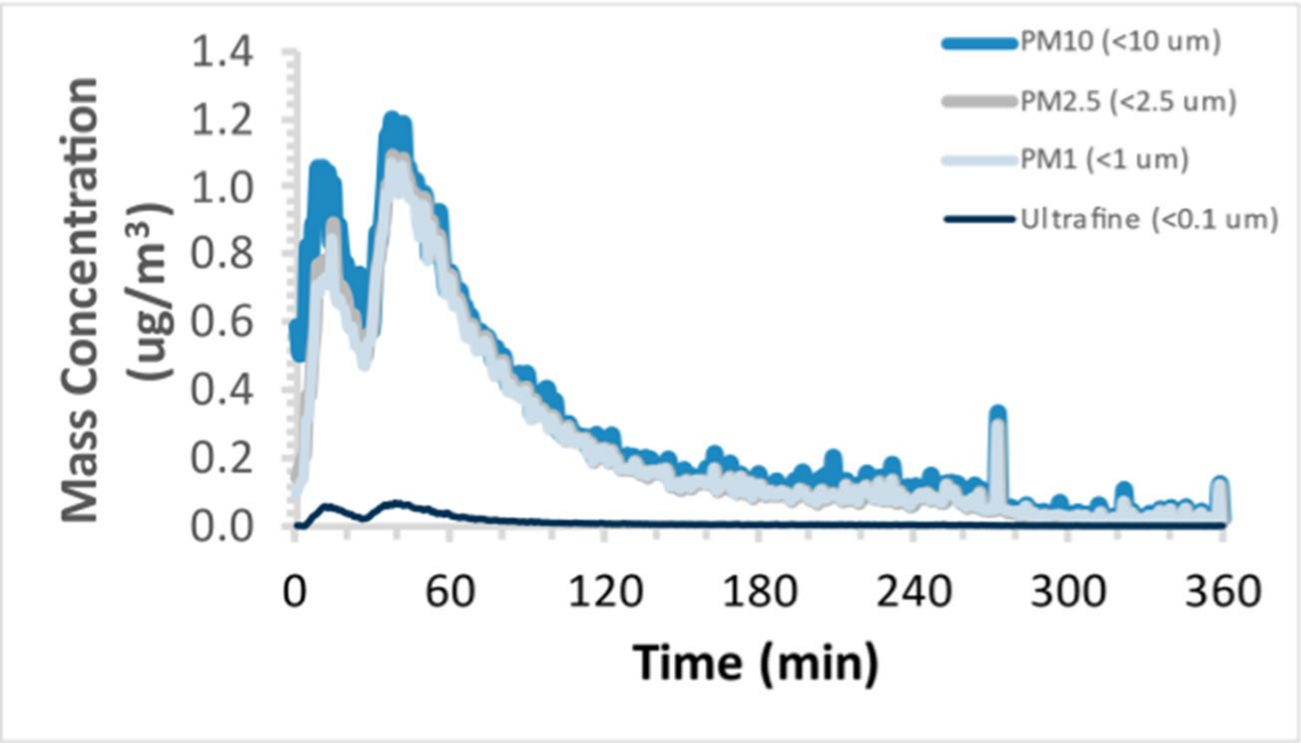
PLA (240°C)	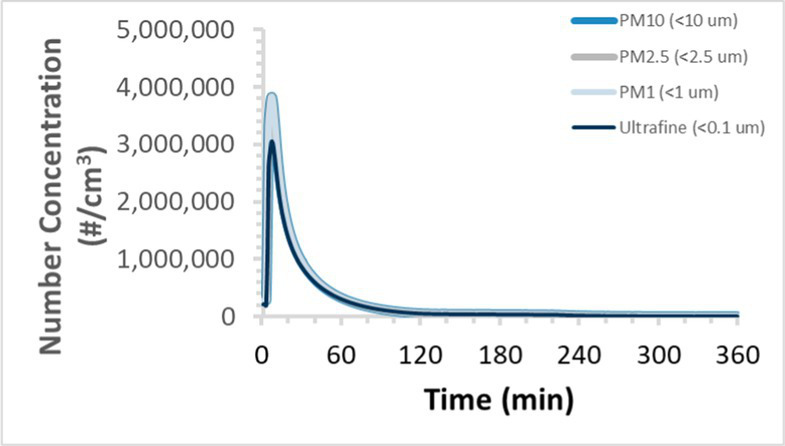	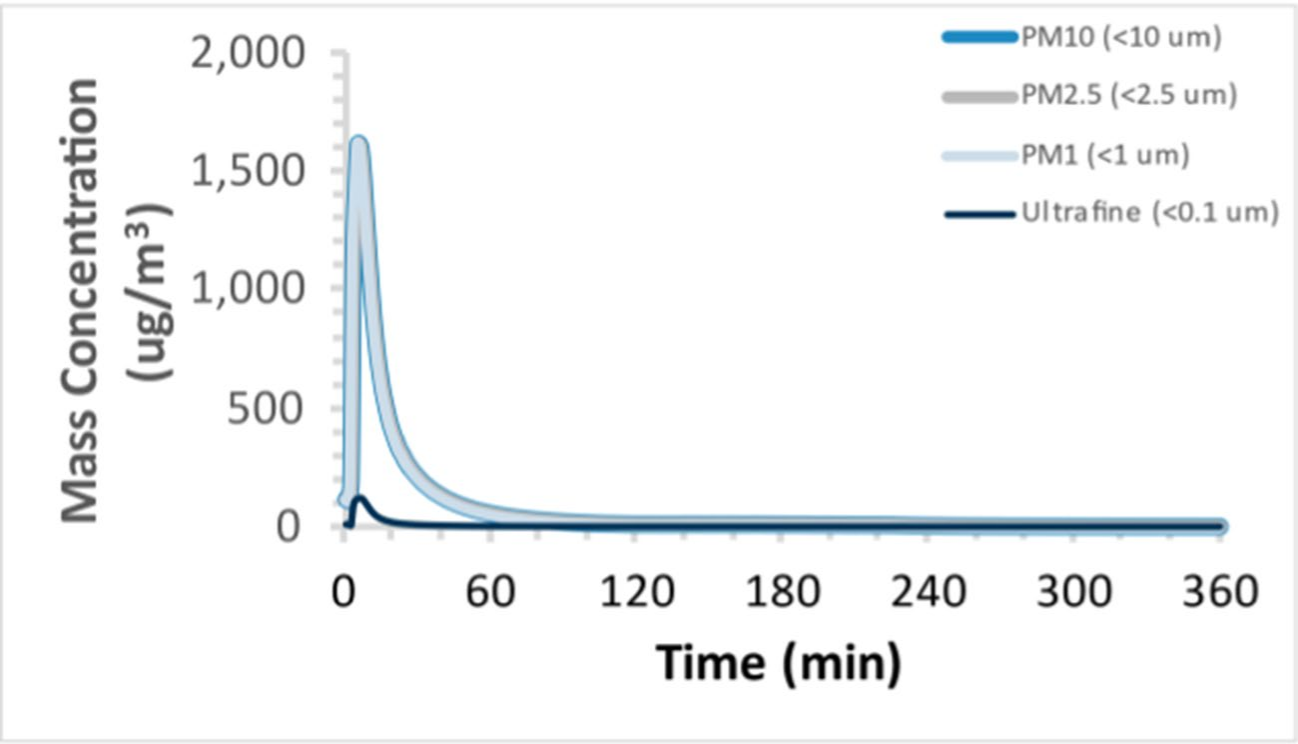
PETG (240°C)	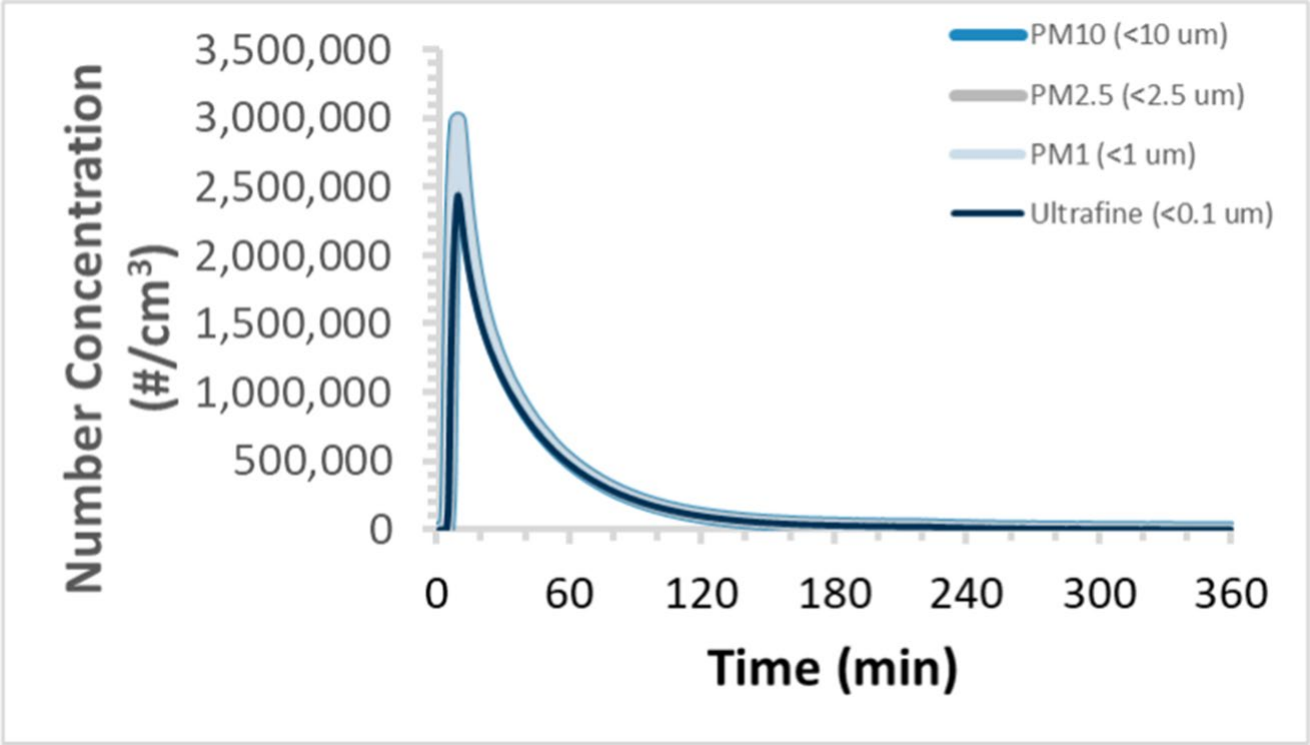	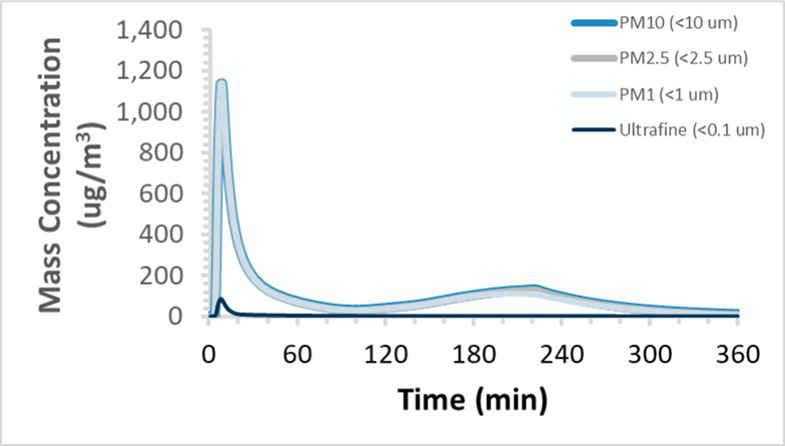
Nylon (260°C)	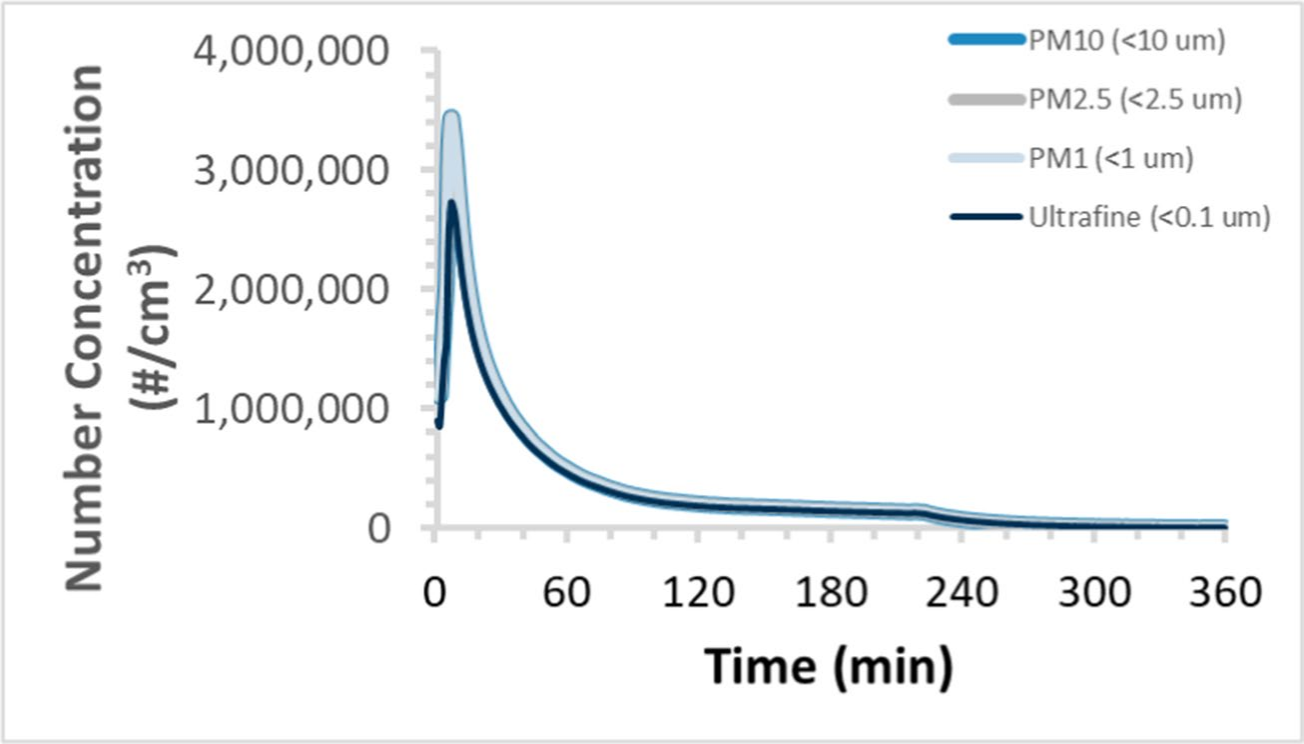	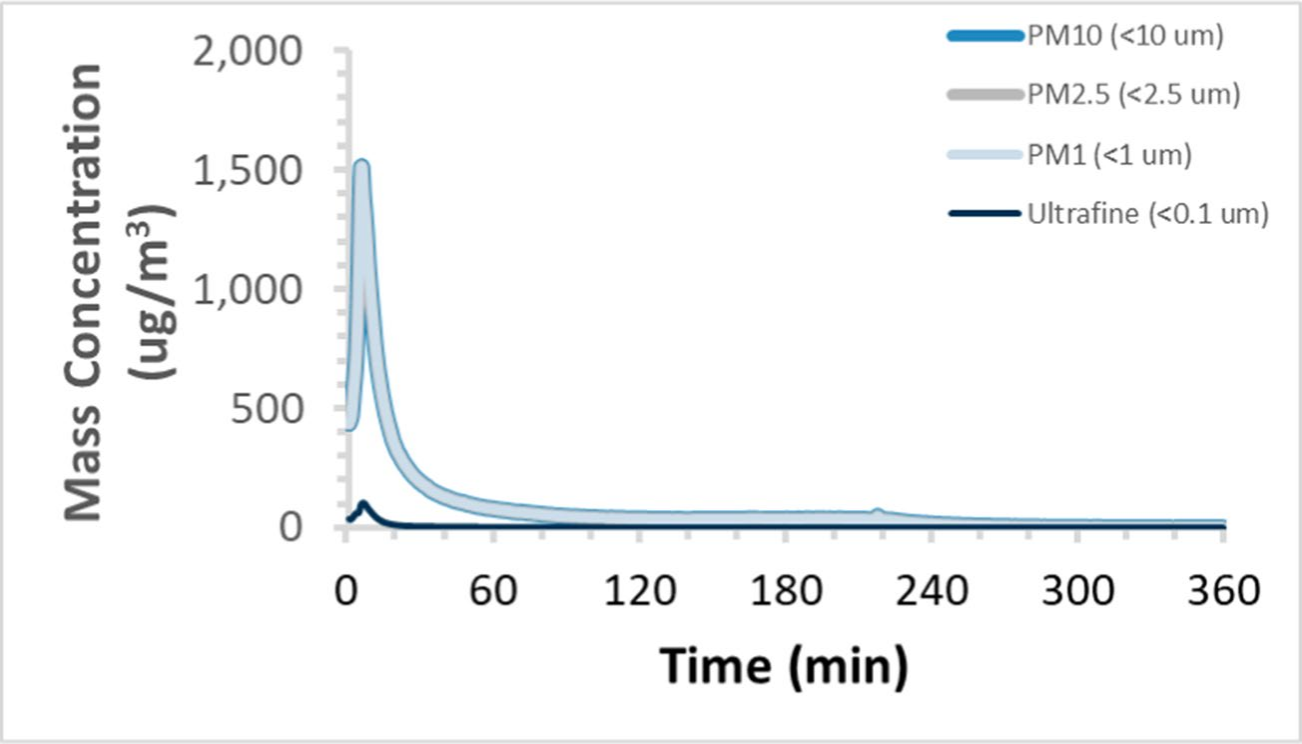
ASA (240°C)	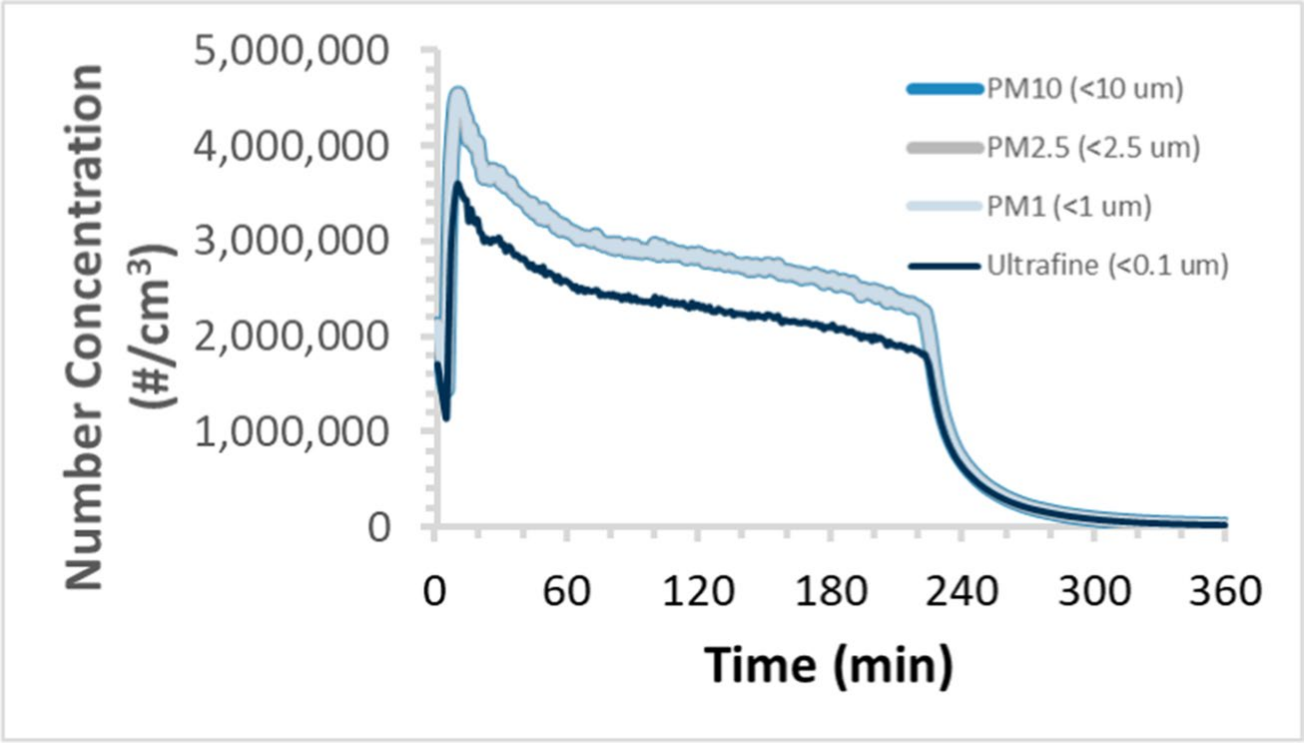	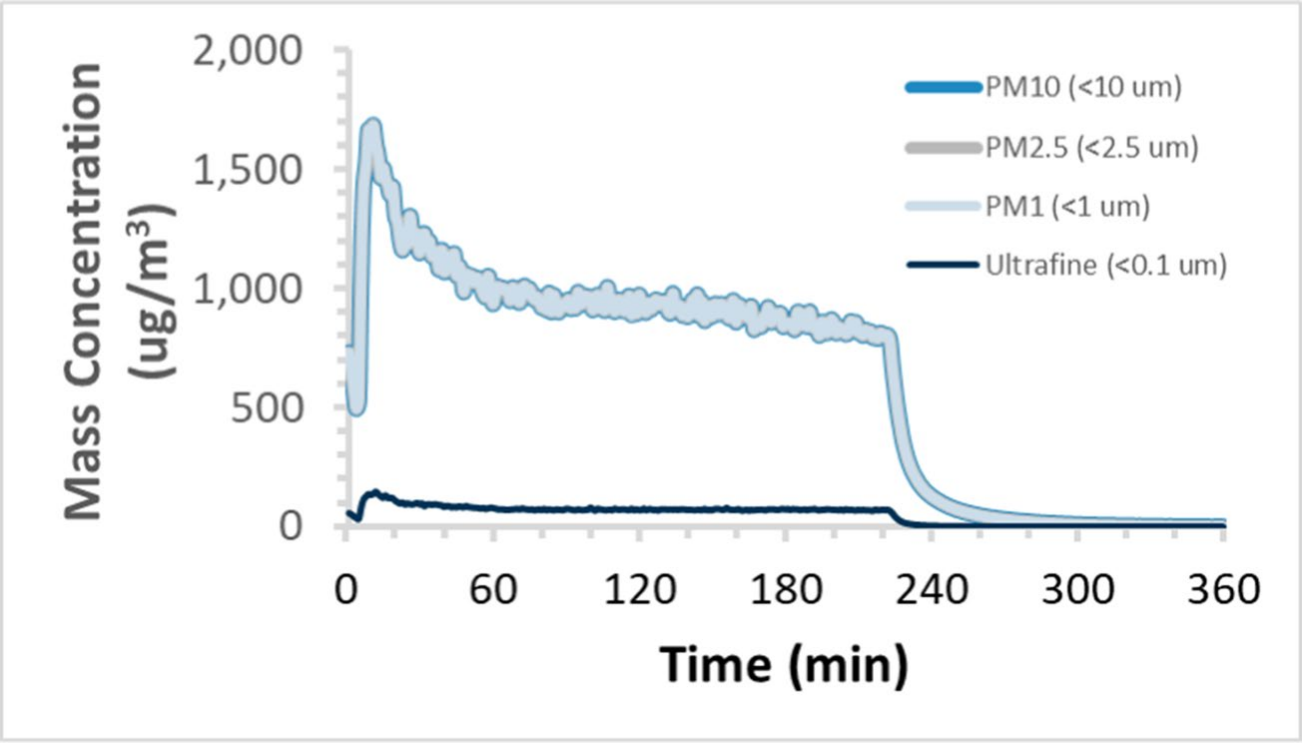

Emissions from PLA at 200°C were below the range of operation stated by the SMPS manufacturer; data are considered advisory.

We demonstrated that the concentration of emitted particulates from 3D printers is not consistent over the duration of the print as measured using the ANSI/CAN/UL 2904 method. Commonly, particulate emissions peak at the beginning of the print and decay to a steady concentration within the first hour which persists for the remainder of the print; the time required to print the 40 mm cube used in this study was approximately 240 min, with data recording continuing for 120 min following the print.

**Figure 1:**
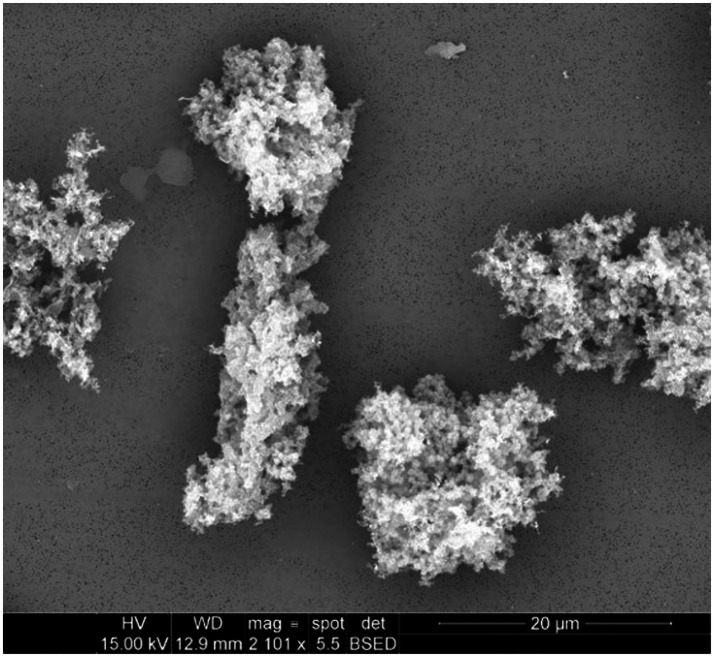
Particulates emitted during printing of ABS at an extrusion temperature of 240°C were captured onto 0.1 μm PCTE membranes. Primary particles were generally submicron and appeared to agglomerate freely.

**Figure 2:**
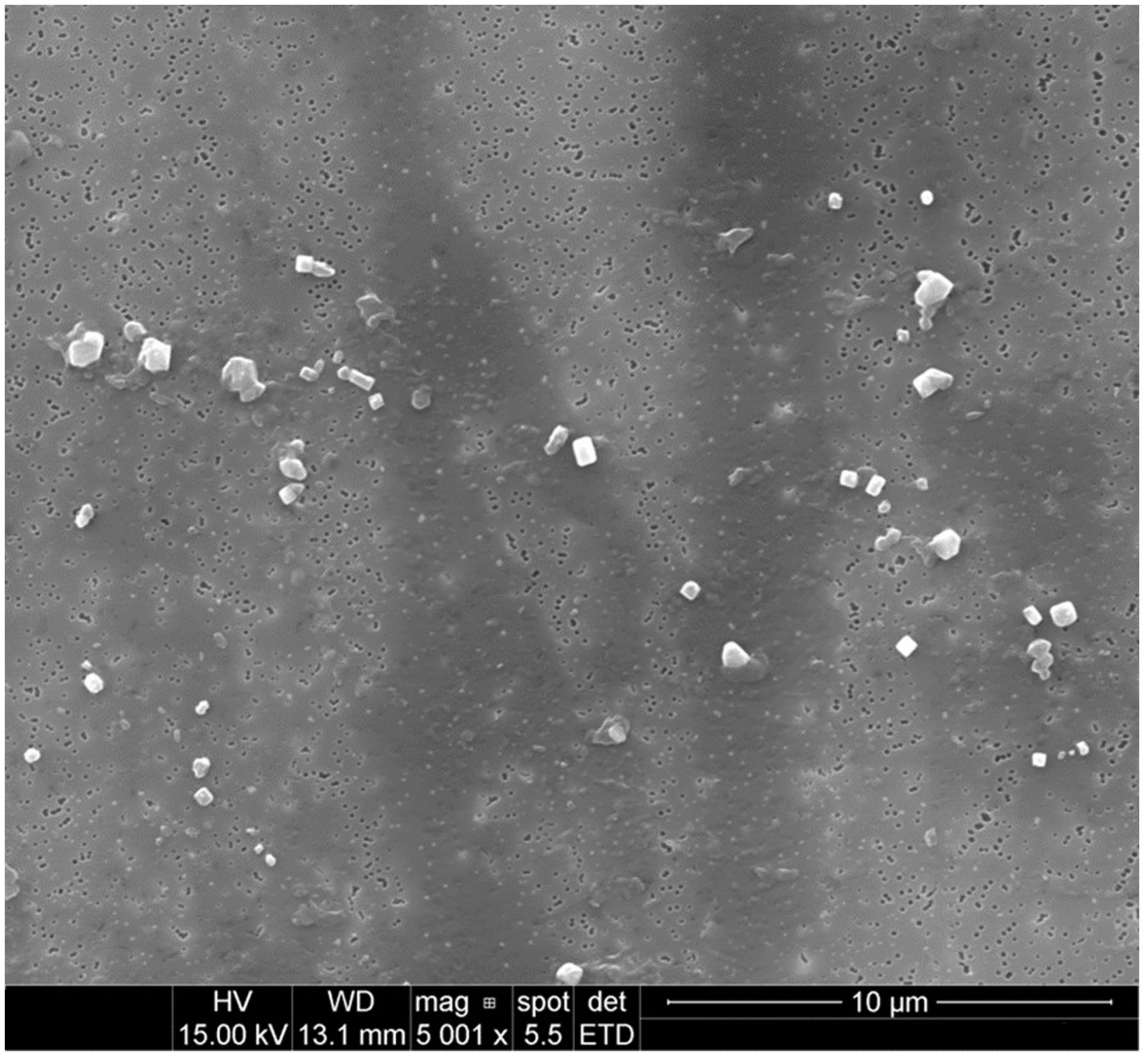
Particulates emitted during printing of PLA at an extrusion temperature of 240°C were captured onto 0.1 μm PCTE membranes. Primary particles did not appear to significantly agglomerate and were predominantly submicron (at this magnification, larger microparticles are more readily apparent above the background of more plentiful submicron particles).

**Figure 3:**
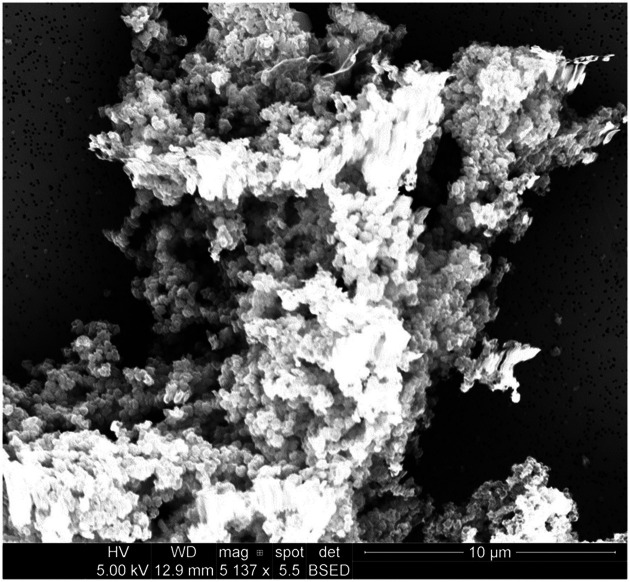
Particulates emitted during printing of PETG at an extrusion temperature of 240°C were captured onto 0.1 μm PCTE membranes. Similar to ABS, primary particles were generally submicron and appeared to agglomerate freely. Charging effects were more difficult to avoid when imaging large agglomerates, causing mild image distortion.

**Figure 4:**
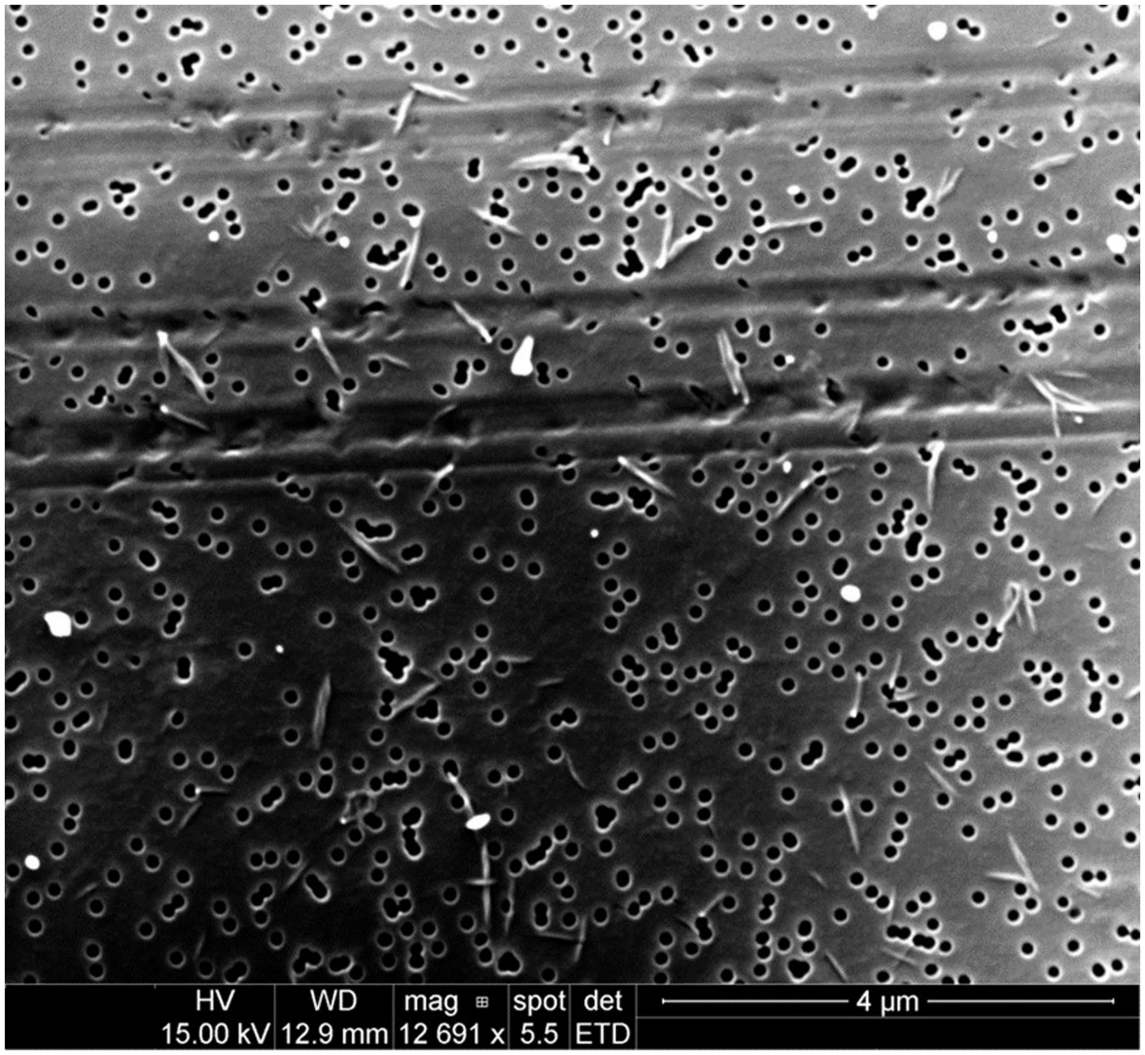
Particulates emitted during printing of nylon at an extrusion temperature of 260°C were captured onto 0.1 μm PCTE membranes. Significantly, most emitted particles were not spheroidal as had been typical of emissions from other filaments, but were instead of a higher aspect ratio (10:1; the short, fibrous particulates measure approximately 60 × 600 nm).

Emitted particulates were sampled onto PCTE membranes for morphological analysis by SEM. Representative images for several filaments are presented in Figures 1–4.

**Figure 5:**
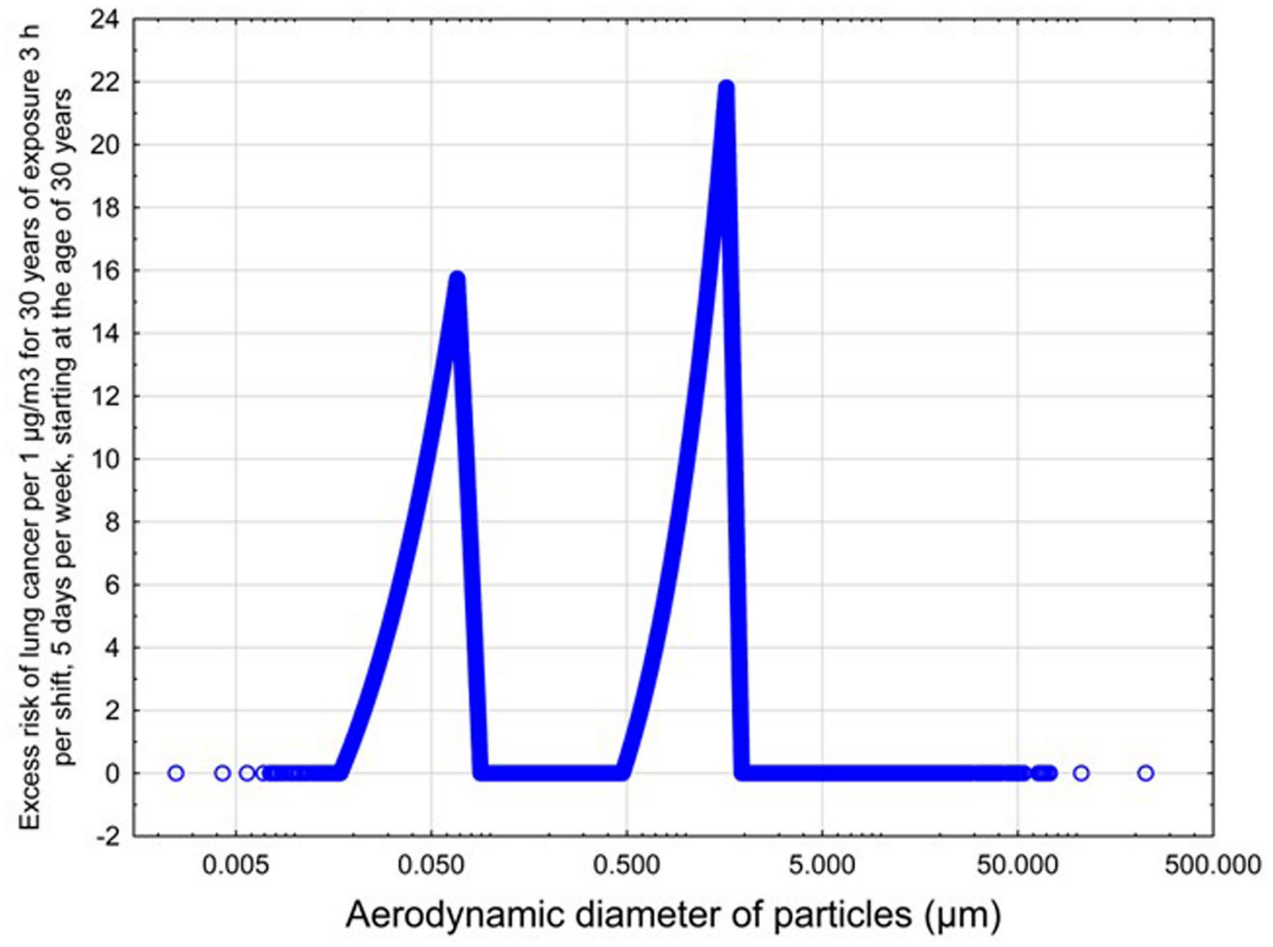
Predicted risk of lungs cancer as a function of aerodynamic diameter for non-elongate particles.

The predicted excess lung cancer risk is highly dependent upon the size distribution of the emitted particulates. The relationship between lung cancer risk per 1 μg/m^3^, assuming 30 years of exposure, 3 h per shift, starting at the age of 30 years, and aerodynamic diameter is illustrated below in Figure 5, based on the model developed in our previous work (38).

The measured size distribution of particulates emitted during 3D printing varied with filament type and extrusion temperature. Mass fraction corresponding to aerodynamic diameter (representable by normalized size distribution) significantly affects the predicted carcinogenic potential; the excess lung cancer risk reported in Table 2 was calculated based upon the measured size distributions that are illustrated in Table 3.

**Table 2:** Excess lung cancer risk is predicted by the model based on the measured particulate distribution.

Filament and extrusion temperature	Excess lung cancer risk (per 10,000 workers, per 1 μg/m^3^)	Average stable mass concentration during print (μg/m^3^)	Excess lung cancer risk based upon exposure to average stable mass concentration (per 10,000 workers)
ABS (220°C)	0.017	3.67	1.44
ABS (230°C)	0.324	18.2	5.89
ABS (240°C)	0.742	192	143
PLA (200°C)	0.704	0.060	0.042
PLA (220°C)	0.854	0.058	0.049
PLA (230°C)	0.604	0.165	0.100
PLA (240°C)	0.245	9.46	2.32
PETG (240°C)	1.47	70.1	103
Nylon (260°C)	0.205	37.2	7.63
ASA (240°C)	0.516	906	468

**Table 3:** Normalized size distribution (mass fraction versus aerodynamic diameter) of emitted particulates measured during 3D printing of various filaments and extrusion temperatures.

Filament and extrusion temperature	Normalized size distribution
ABS (220°C)	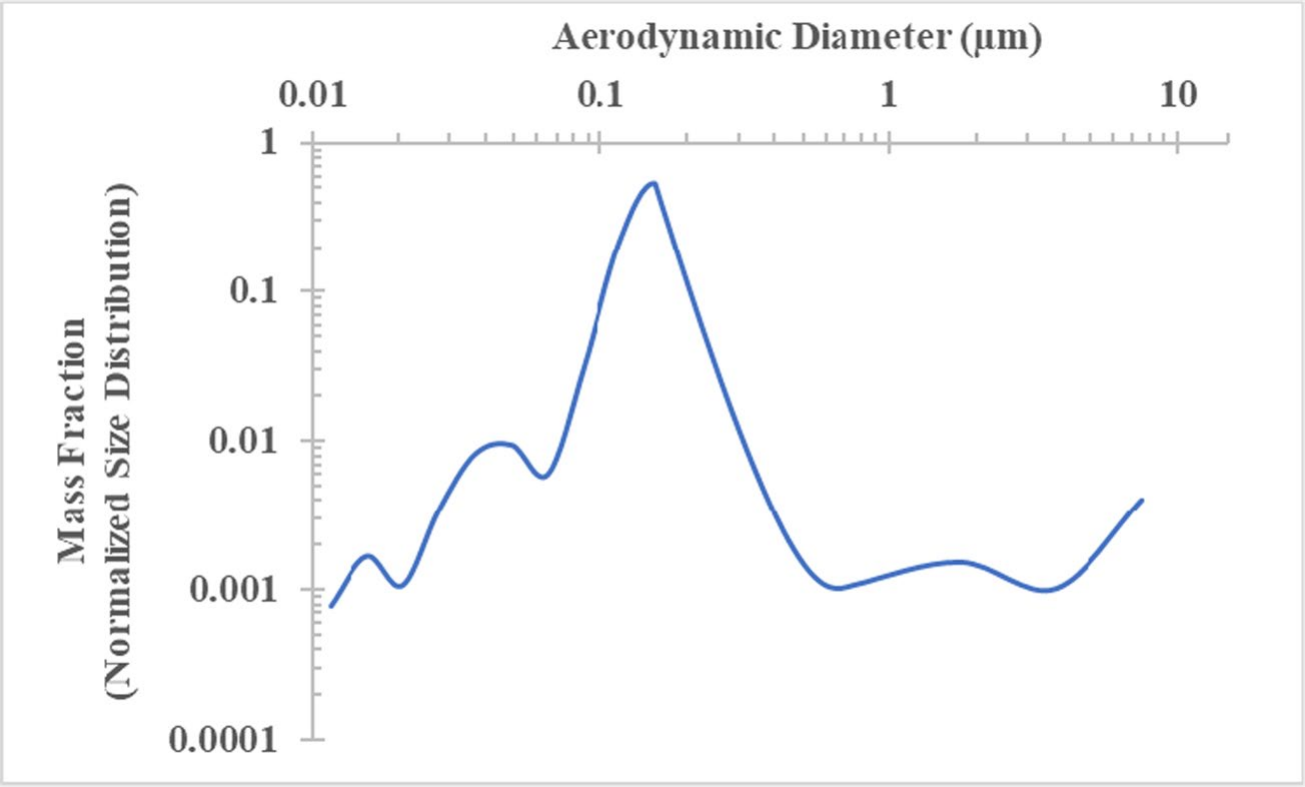
ABS (230°C)	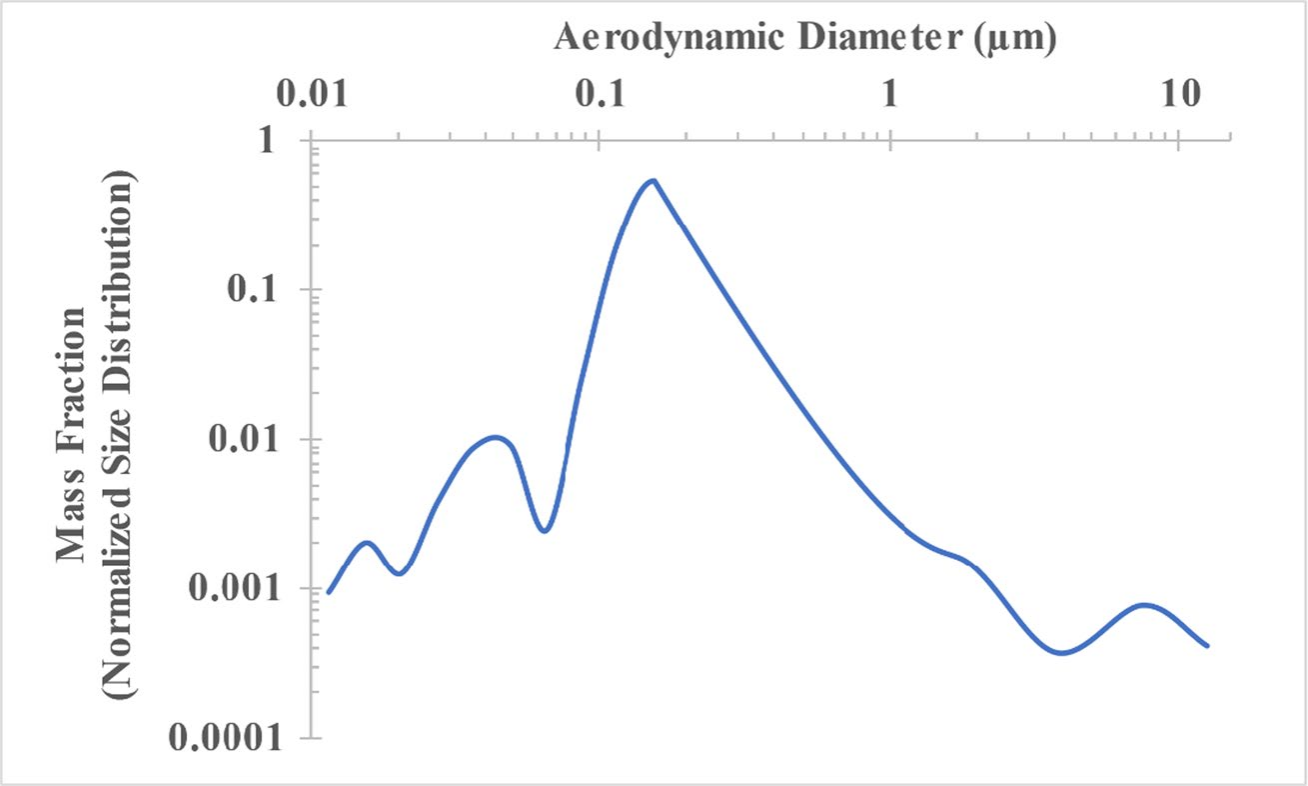
ABS (240°C)	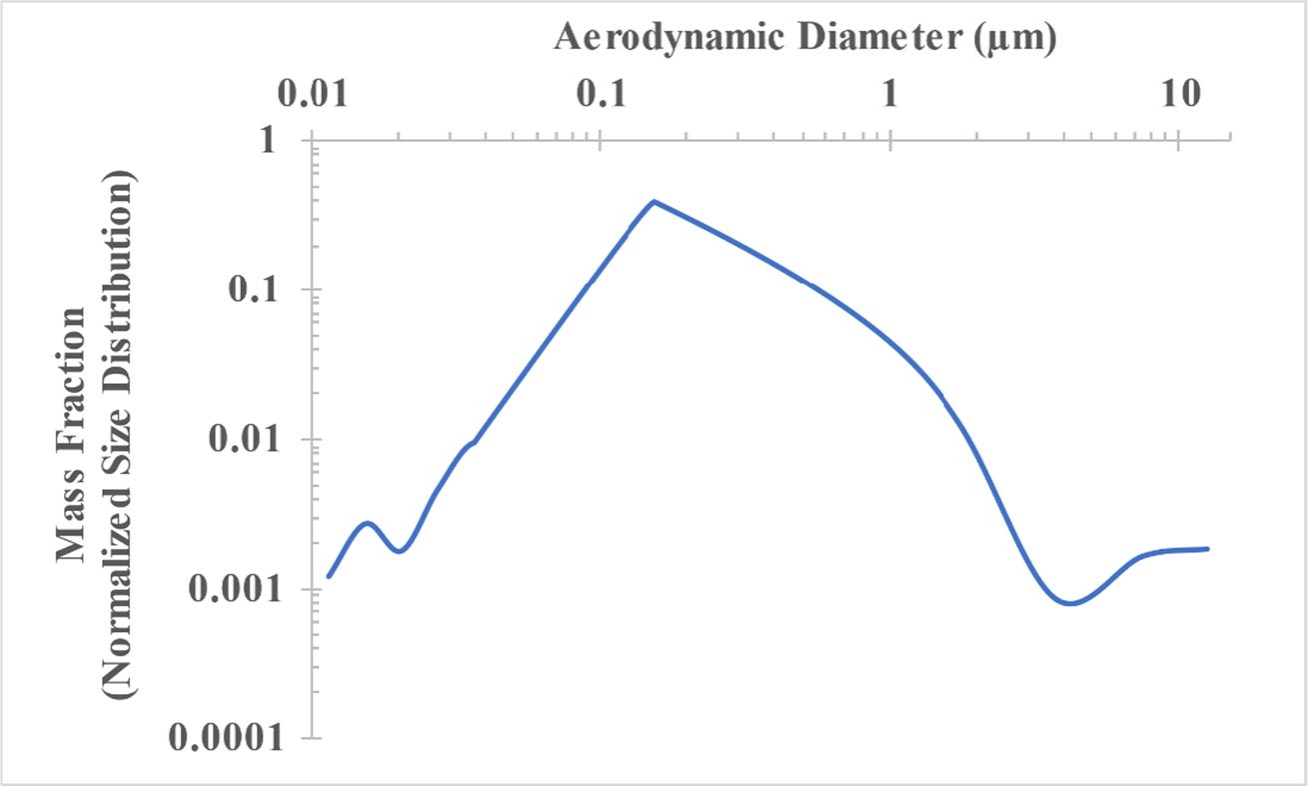
PLA (200°C)^1^	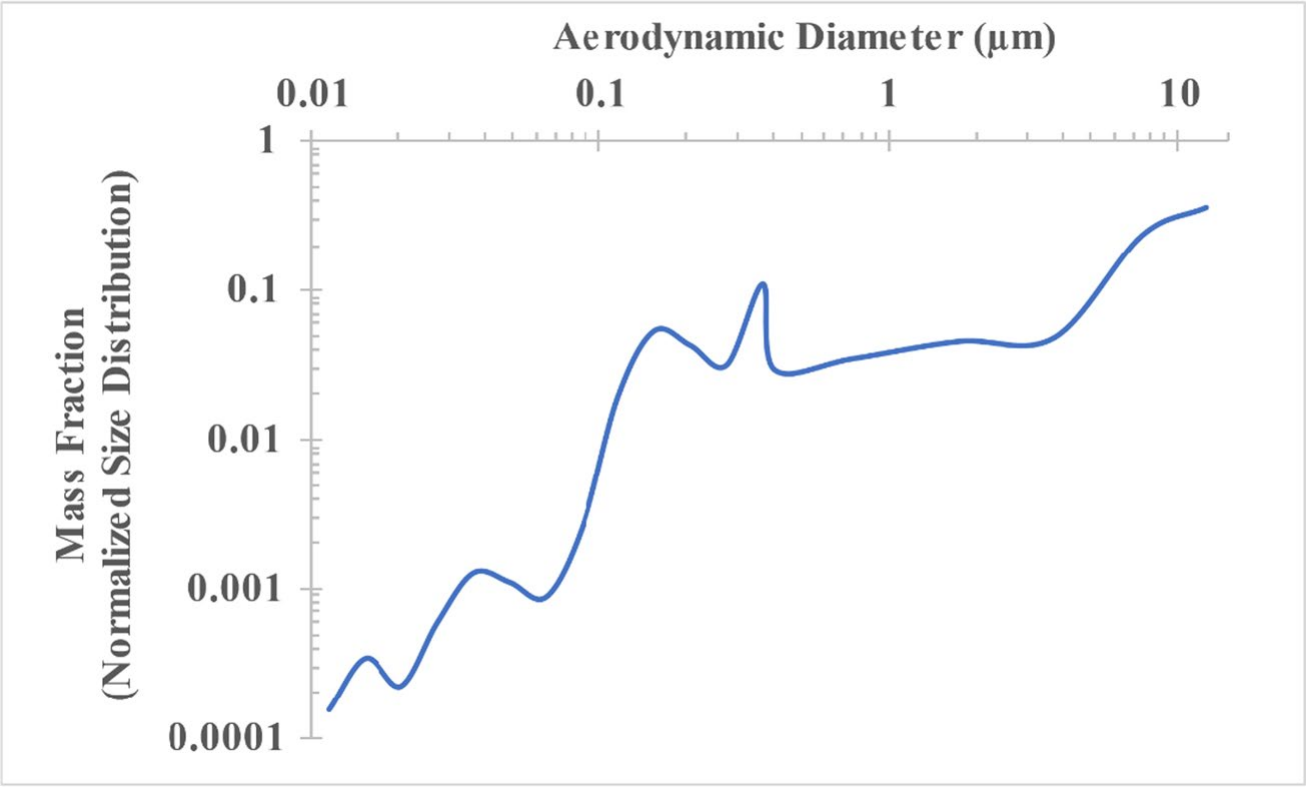
PLA (220°C)	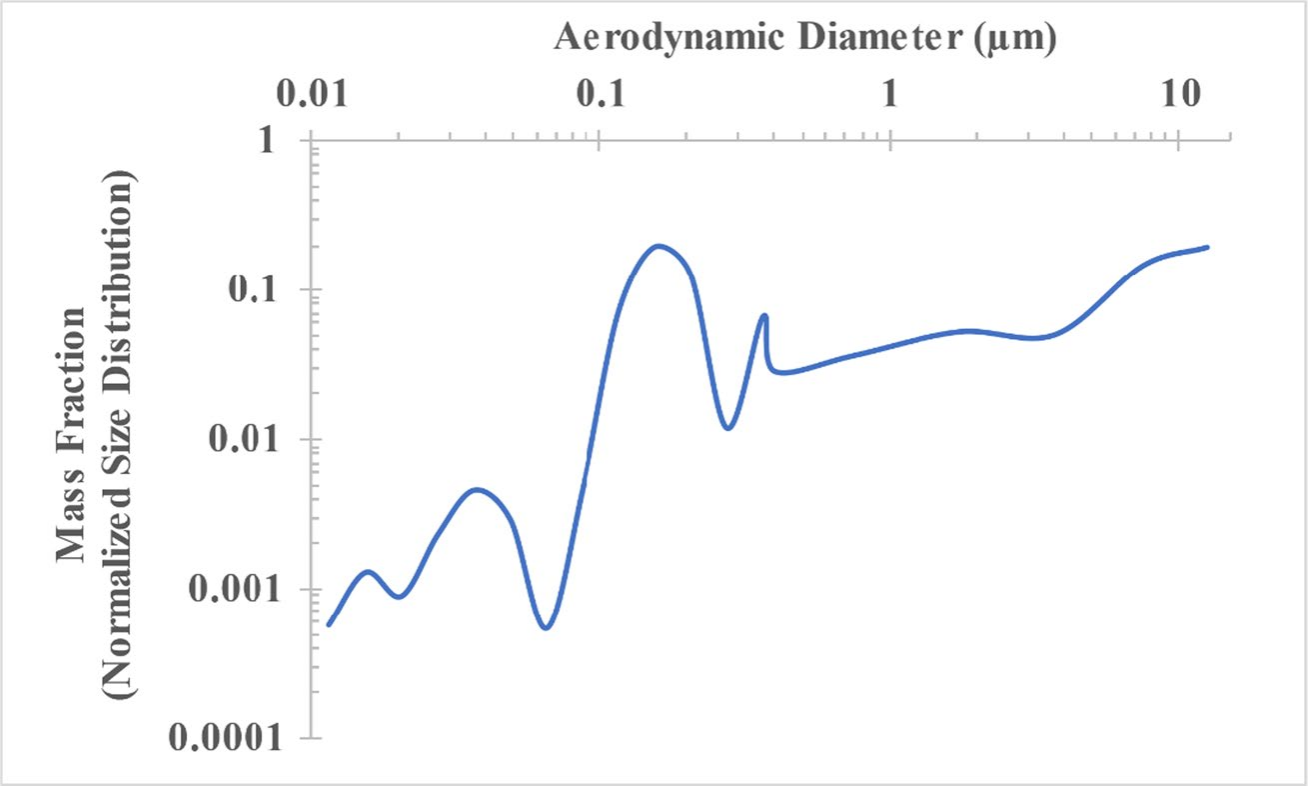
PLA (230°C)	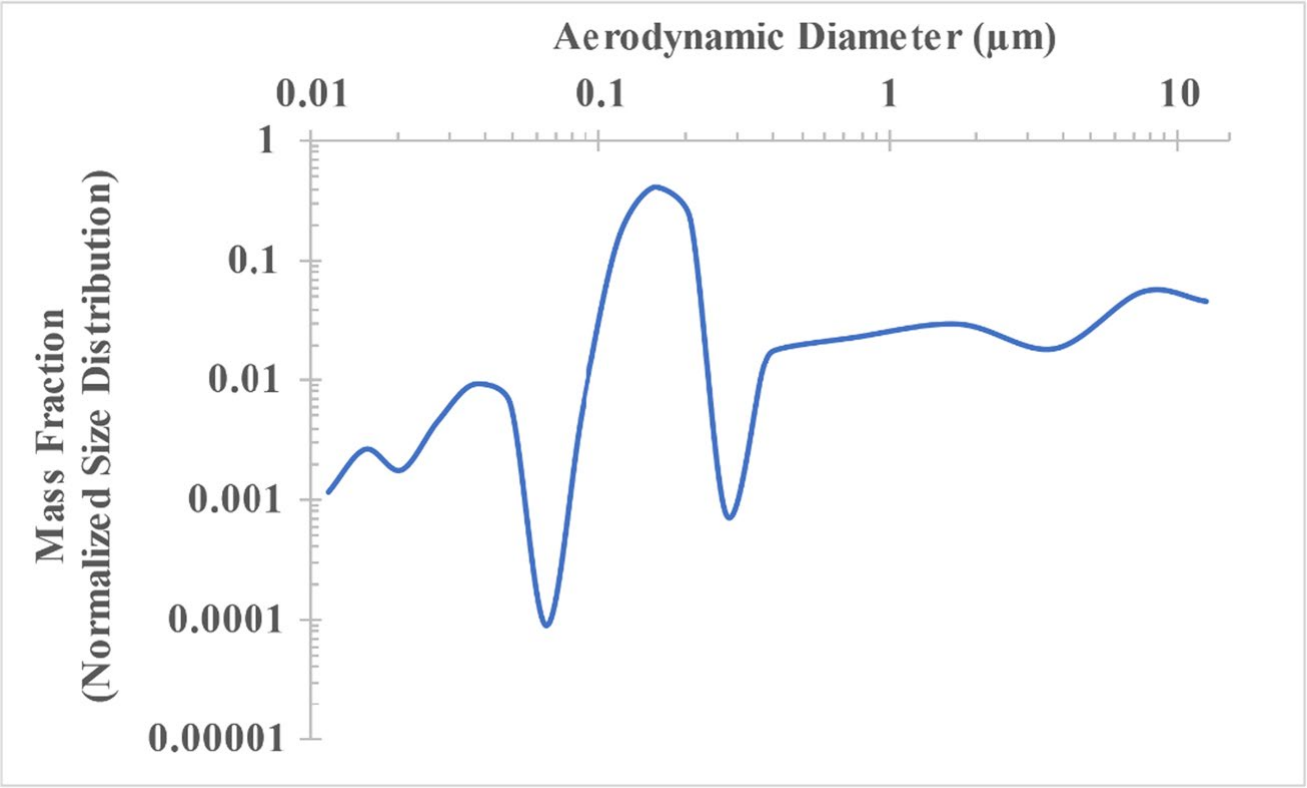
PLA (240°C)	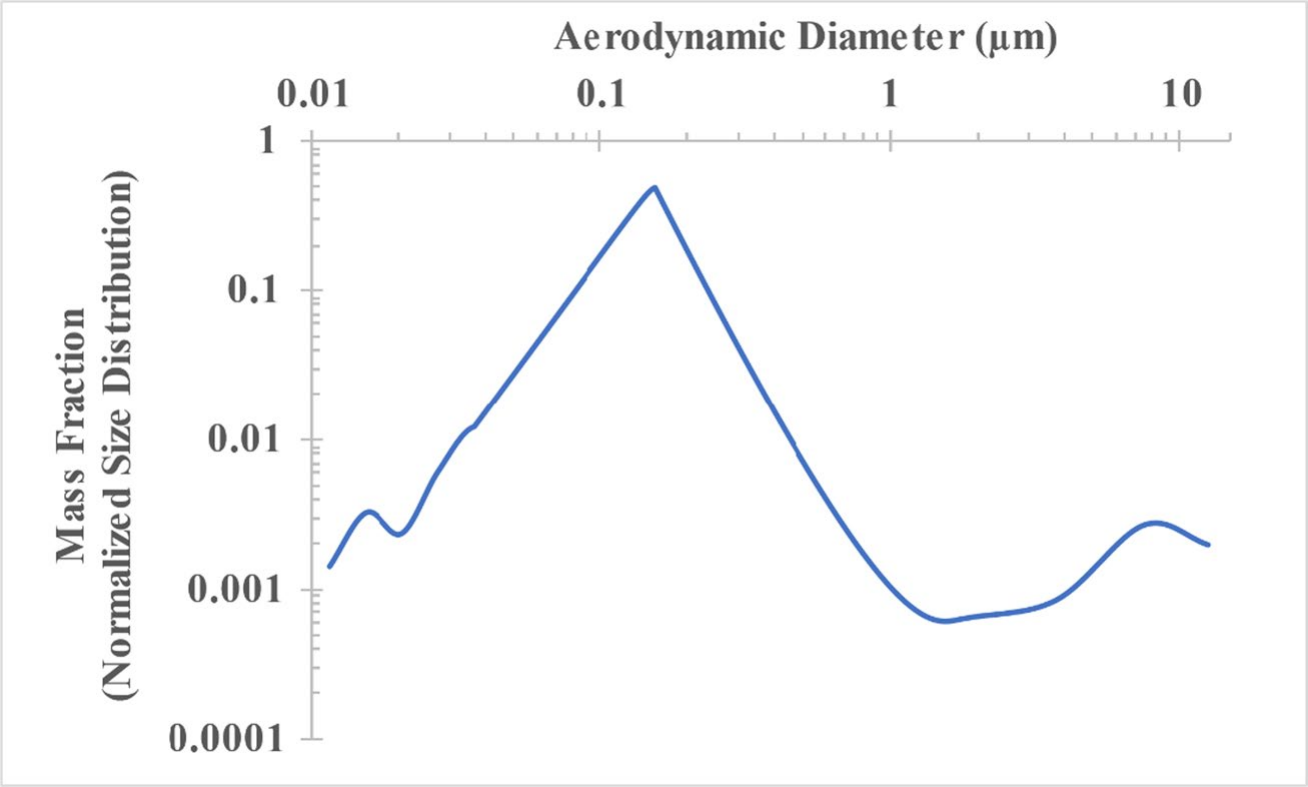
PETG (240°C)	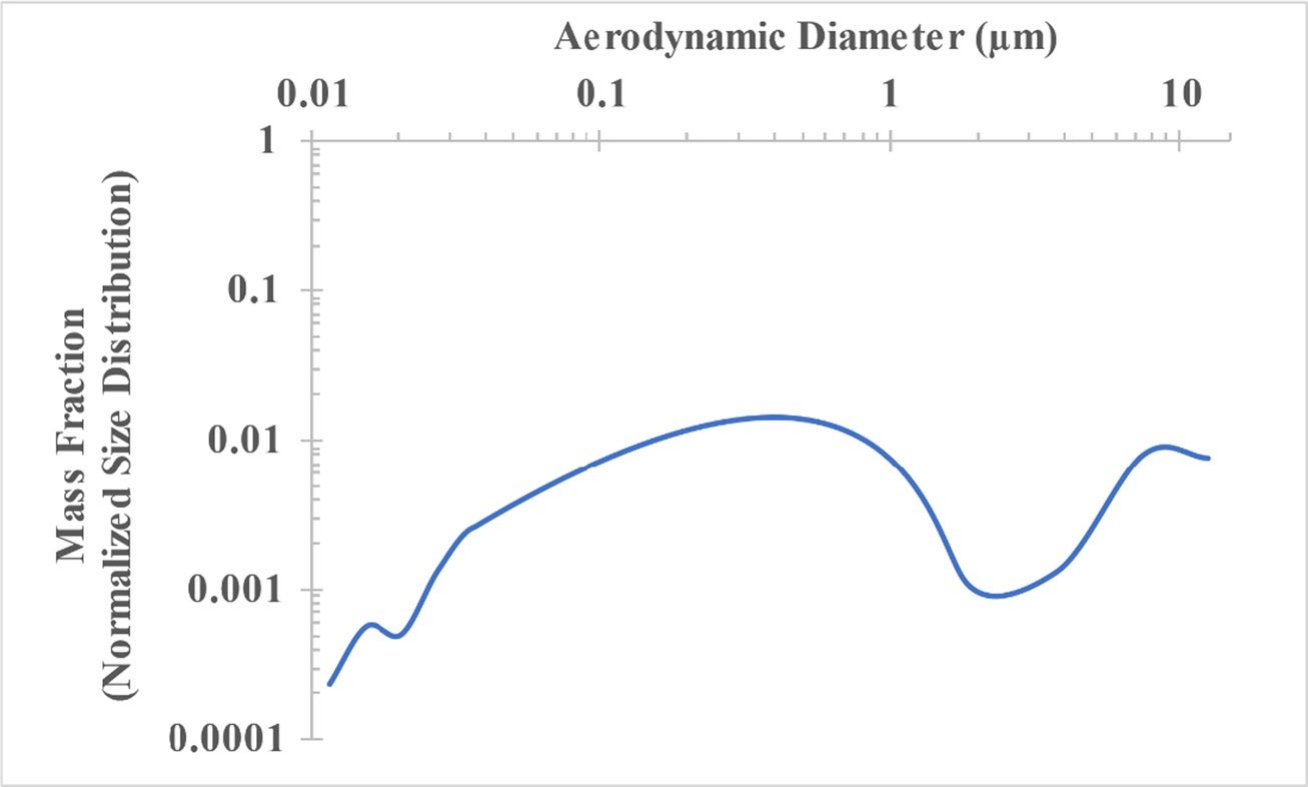
Nylon (260°C)	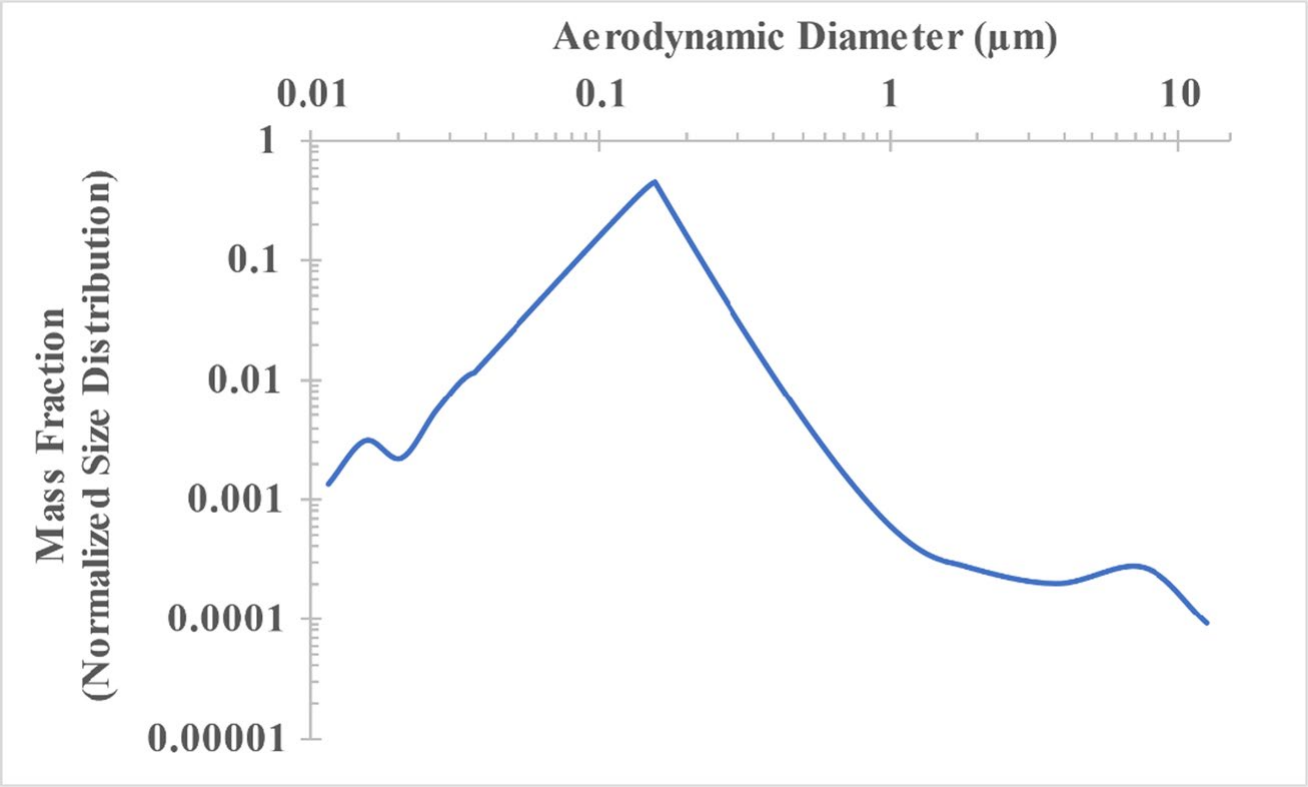
ASA (240°C)	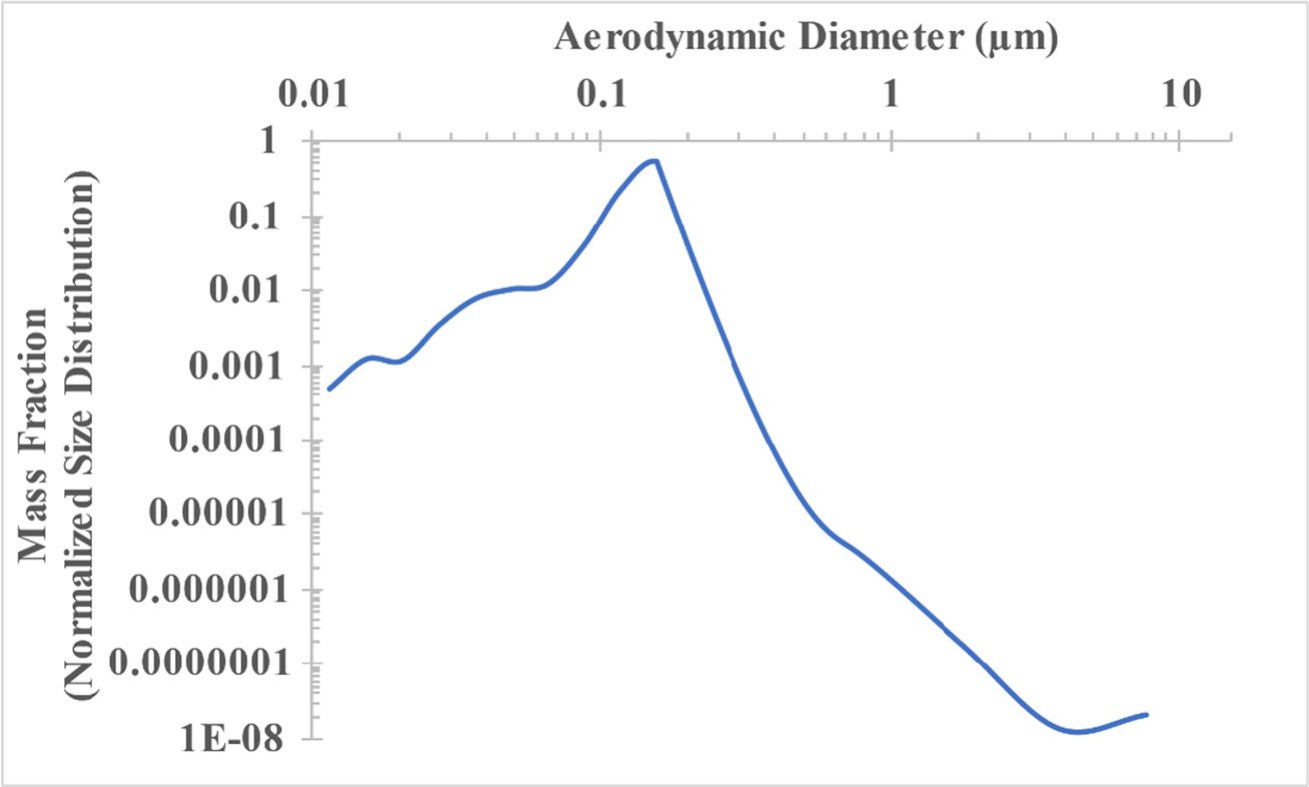

1 Emissions from PLA at 200°C were below the range of operation stated by the SMPS manufacturer; data are considered advisory.

The developed model was used to predict the excess lung cancer risk presented by the measured emission profiles based upon concentration and size distribution. As most prints exhibited a substantial “peak” emission early in the print whose concentration was significantly elevated in comparison to the “stable” emission observed during the remainder of the print, modeled values are calculated based on the “stable” concentration. Integration of “peak” emissions into the calculations was considered; multiple parameters including print duration, print quantity during a shift, number of printers operating simultaneously, and room characteristics will impact the contribution of peak emissions to the total exposure profile. Accordingly, the data presented here are considered to be exemplary and advisory while only “stable” concentration values are used as inputs, with further enhancements to the model in future work to enable input of “peak” values in the context of print and environmental conditions.

## 4 Discussion

### 4.1 Emission concentration characteristics

Upon reviewing the particulate emission data, several observations are worth noting specifically which bear importance upon the modeled excess lung cancer risk.

Universally, printing at lower extrusion temperature reduces the emitted particulate concentrations, as observed in Table 1. In the conditions of this study, for example, increasing extrusion temperature of ABS by 10°C resulted in an order of magnitude increase in peak emissions; mass concentration peaked around 8 μg/cm^3^ when printing at 220°C but rose to nearly 80 μg/cm^3^ at 230°C and over 1,200 μg/cm^3^ at 240°C.

Similarly, printing PLA near its lowest recommended extrusion temperature (200°C) generally reduced released emission concentrations to less than 100 #/cm^3^, which is the stated reliable operational limit by the manufacturer of the SMPS used in this study (as indicated, perhaps, by the noise spikes evident in the profile in Table 1). Accordingly, this particular set of data (PLA at 200°C) is considered advisory but is included to demonstrate the trend of decreasing emissions with decreasing extrusion temperature.

Further, it is important to note that manufacturers typically do not recommend printing PLA above 220°C, and prints carried out at 230°C and 240°C were conducted for comparison (the 40 mm cube was printable at these temperatures, but more detailed models may experience unfavorable deformations in these conditions). Increasing PLA extrusion temperature to 240°C increased its peak emissions to the same order of magnitude as ABS, PETG, Nylon, and ASA, but, importantly, its sustained, stable emissions after the initial peak remained relatively low in comparison to these other filaments.

### 4.2 Emitted particulate morphology

Emitted particulates are assumed by the instruments to be spheroidal, which generally appeared to hold true based upon morphological observations provided by SEM imaging in Figures 1–3 (nylon, Figure 4, being the notable exception, as discussed later). In agreement with the SMPS and OPC measurements, the vast majority of captured particulates appeared to exhibit primary particle diameters that were submicron. However, agglomerative behavior was observed in the case of ABS and PETG which may complicate health impacts; as noted in previous work (19), the primary emitted particle size does not appear to change discernibly over the course of the print, but agglomeration may occur which could affect lung deposition (and later release if agglomerates dissociate after deposition). An unfavorable outcome might be that agglomerates increase lung deposition while increasing total ultrafine particle loading by preventing exhalation of nanoparticles which can later dissociate from the aggregates. This comment is hypothetical and requires additional, detailed study regarding deposition and dissociation which is considered outside the scope of this study. It is also not definitively certain whether the observed agglomeration occurs in-air or on the membrane substrate over the course of the sampling duration, though as we have suggested previously (19), in-air agglomeration may be indicated by the real-time characterization data, as the ultrafine emission peak decreases in tandem with an increase in the concentration larger “particles” reported by the equipment later in the print, yet SEM imaging does not observe matching primary particles of larger diameter (suggesting that these larger agglomerates may instead be counted by the OPC).

Natural PLA was not observed to exhibit the same agglomerative behavior, though it was observed in a previous study that additives in the filament (specifically, carbon nanotubes used for static dissipation) could induce agglomerative formations similar to those observed during printing of ABS and PETG (19).

Concerningly, nylon was observed to emit particulates of relatively high aspect ratio in comparison to the more spheroidal particulates captured during printing of ABS, PETG, and PLA (particulates emitted during printing of ASA were not analyzed by SEM). Nylon emissions pictured in Figure 4 contained particulates that were of consistent size and morphology, measuring approximately 60 nm × 600 nm (aspect ratio of 10:1). Asbestiform fibers generally exhibit aspect ratios of 20:1 or higher (48); emitted nylon particulates are therefore not directly comparable but their elongated form may yet present additional concern to be analyzed further in future studies.

### 4.3 Emitted particulate size distribution (aerodynamic diameter)

The dependence of predicted carcinogenic potential on aerodynamic diameter as developed in our previous work (38) is illustrated in Figure 5. Nearly all tested filaments exhibited aerodynamic diameter distributions that peaked between 0.1 and 0.2 μm, which happens to fall near the second minima of the predicted carcinogenic potential. Accordingly, the modeled carcinogenic potential is reduced for filaments exhibiting a sharper peak in this region. The aerodynamic diameter distribution expands in breadth as extrusion temperature increases during printing of ABS, and the distribution is especially broad in the case of PETG, causing each to exhibit a wider range of aerodynamic diameters which contribute more strongly to the carcinogenic potential predicted by our model.

### 4.4 Modeled excess lung cancer risk

Predicted carcinogenic potential is dependent upon emitted aerodynamic diameter in accordance with the bimodal relationship pictured in Figure 5; as noted previously, most filaments exhibited aerodynamic diameter distribution maxima near the minima for predicted carcinogenic potential. The qualitative breadth of the aerodynamic distribution therefore became a strong predictor for carcinogenic potential.

The aerodynamic diameter distribution of ABS expanded in breadth with increasing extrusion temperature, resulting in an increase in modeled excess lung cancer risk (per 10,000 workers per 1 μg/m^3^) from 0.017 for ABS printed at 220°C to 0.324 and 0.742 for prints at extrusion temperatures of 230°C and 240°C, respectively. PETG (printed at its recommended extrusion temperature of 240°C) exhibited the broadest aerodynamic diameter distribution observed in this study; correspondingly, its modeled excess lung cancer risk per 10,000 workers per 1 μg/m^3^ was the highest of all tested filaments (1.47).

The base carcinogenic potential and modeled excess lung cancer risk per 10,000 workers per 1 μg/m^3^ can be used to assess an exposure-based excess lung cancer risk for a real-life print scenario. Toxicological inferences must consider a range of possible parameters, including the brief peak emission concentration versus stable emission concentration (i.e., typical long-term exposure) and the impacts of deployed conditions. Calculations are based upon particulate concentrations measured by the ANSI/CAN/UL 2904 method (i.e., within the ~1 m^3^ enclosure with an air exchange rate of 1/h), which ensures repeatability of measurements and cross-comparison between filaments and print conditions. However, the particulate exposure experienced by the user will be altered in deployed scenarios as depends upon room volume, air exchange/ventilation/filtration, number of printers operating simultaneously and duration of print, and other factors. Consequently, the modeled excess lung cancer risk would be reduced in rooms with greater air volume or active engineering controls (as particulate concentrations decrease from those measured in this study) or may increase in spaces where many printers are operating at once or which exhibit limited ventilation (as particulate concentrations increase from those measured in this study).

Accordingly, the following estimations should be interpreted in their experimental context; while the emitted size distribution presented here is expected to be applicable across all deployed scenarios, the measured concentration of emitted particulates will vary in accordance with factors such as room size, ventilation, filtration, number of printers operating simultaneously, and, presumably, print duration and frequency (since all filaments exhibit emission peaks early in the print process before stabilizing at a lower emitted concentration, which might result in elevated particulate emissions if shorter, more frequent prints are conducted).

A small room may be expected to contain an air volume of 15 m^3^ or greater, albeit possibly at a lower exchange rate than the 1/h in this study, since the minimum recommended exchange rate in buildings is 0.35/h per ASHRAE (49). Similarly, it is not uncommon for many 3D printers to be operated simultaneously in close proximity, especially in engineering or industrial settings. The dilutive effect of room volume and air exchange (or filtration) should be considered alongside additive effects of the quantity of printers and other print parameter considerations when this model is used in practical settings.

The following inferences are therefore based upon the concentrations of particulates measured within the 1 m^3^ chamber used in this study with an air exchange rate of 1/h. Table 2 describes the excess lung cancer risk presented by the tested scenario using the measured concentrations, which accounts for the modeled carcinogenic potential based upon particulate aerodynamic diameter distribution as well as exposed particulate concentration.

Standard guidance from manufacturers is generally that ABS may be printed at extrusion temperatures of 220–240°C. This work makes a strong case for printing at the lowest extrusion temperature possible that results in acceptable print quality, as the excess lung cancer risk presented during printing of ABS increases from 1.44 per 10,000 workers at 220°C to 143 per 10,000 workers at 240°C when aerodynamic diameter distribution and measured emitted concentration are considered together. Similar trends are observed during printing of PLA (excess lung cancer risk per 10,000 workers increases from 0.042 at 200°C to 2.32 at 240°C), but the overall impact is limited since the stable emission concentration of PLA remains relatively low in comparison to other filaments.

Nylon exhibited a relatively low risk per 10,000 workers per 1 μg/m^3^ (0.205) and moderate stable emitted concentration (37.2 μg/m^3^), resulting in a modeled excess lung cancer risk of 7.63 per 10,000 workers in the measured scenario. However, this estimation assumes spheroidal particles; as nylon emissions were shown previously (Figure 4) to be of higher aspect ratio (10:1), health effects may differ from those estimated by this model.

PETG exhibited an excess lung cancer risk of 103 per 10,000 workers; its excess lung cancer risk per 1 μg/m^3^ was the highest among tested filaments but its stable emitted particulate concentration was moderate in comparison to the highest emitters. The excess lung cancer risk per 10,000 workers per 1 μg/m^3^ calculated for ASA (0.516) was moderate in comparison to other filaments at the same extrusion temperature of 240°C (0.742 for ABS and 1.47 for PETG), but ASA recorded the highest stable emission concentration of all tested filaments—906 μg/m^3^ compared with 192 μg/m^3^ for ABS or 70.1 μg/m^3^ for PETG—causing ASA to exhibit the highest estimated excess lung cancer risk of 468 per 10,000 workers of any of the tested filaments.

For context, the excess lung cancer risk presented by carbon nanotubes per 10,000 workers per 1 μg/m^3^ is estimated to be 5.45 (50). Since NIOSH has established a recommended exposure limit (REL) of 1 μg/m^3^ for CNTs (51), this effectively represents an accepted excess lung cancer risk of 5.45 per 10,000 workers. The NIOSH REL for crystalline silica (as respirable dust) is currently set at 50 μg/m^3^, and the excess lung cancer risk per 10,000 workers per 1 μg/m^3^ has been defined as 1.7 (52), suggesting an exposure-based excess lung cancer risk of 85 per 10,000 workers per 1 μg/m^3^.

Accordingly, the excess lung cancer risk per 10,000 workers presented by ABS, PETG, and ASA, when printed at an extrusion temperature of 240°C, are shown to exceed that of CNTs or crystalline silica at their respective RELs—5.45 and 85 per 10,000 workers, respectively, for CNTs and crystalline silica based on this model versus 143, 103, and 468 per 10,000 workers, respectively, for ABS, PETG, and ASA at the emission concentrations measured in this work.

To reiterate, the concentrations measured within this work were conducted in a 1 m^3^ chamber in accordance with ANSI/CAN/UL 2904 and are not predictive of the exposure a user will experience in deployed settings but do represent plausible exposure concentrations where room ventilation is limited and multiple printers are operating simultaneously. Even so, significant excess lung cancer risk is predicted to be associated with 3D printer emissions using experimental data; great care should therefore be exercised to ensure adequate room ventilation and filtration, especially when printing filaments at elevated extrusion temperatures.

Our study has uncertainties and limitations. Our analysis of lung cancer risk was based on a conditional assumption that dimensions of non-elongate fine and ultrafine particles can be predictive of potency factors. In reality, there are many other parameters that should be used for modeling of lung cancer risk. In our future studies we plan to develop a databank of particle characteristics to include the modeling of toxicological potential. Further analysis of morphology for particulates emitted by 3D printers is needed to elucidate the input of particle shape (and not just size) in the cancer risk variability. Our study considers carcinogenic risk as depends heavily on particle size and concentration and only referentially includes compositional impacts. This limitation should be plainly noted; as compositional details become available (as corresponds to 3D printer emissions or any other field in which this model may be applied), integration of compositional effects are strongly encouraged.

Additionally, the model leverages the “stable” particulate emission concentration as an input, ignoring the shorter (but often large) “peak” emission that occurs near the beginning of most prints. As discussed, many factors such as print duration, number of prints conducted during a working shift, number of printers operating simultaneously, or room volume/ventilation can impact the integration of peak emission characteristics. These parameters are calculable; as our model is improved, such inputs will be addressed (and validated experimentally) in future work.

Our research demonstrates that emission of 3D printers should be a matter of risk assessment and management. Reduction of particulate emission should be one of the priorities for manufacturers. Increasingly, newer 3D printer models are designed by manufacturers to be fully enclosed and many include integrated particulate and vapor filters; we strongly encourage use of enclosed and filtered printers where possible to reduce exposure risk.

## Data Availability

The raw data supporting the conclusions of this article will be made available by the authors without undue reservation.
